# Structural Genomics Defines PBRM1 Bromodomain Variant Function in ccRCC

**DOI:** 10.1155/humu/9214621

**Published:** 2026-08-05

**Authors:** Karina L. Bursch, Salomão D. Jorge, Audrey E. Catlin, Raymundo Nuñez, Michael D. LeClaire, Jacob Licklider, Raul A. Urrutia, Brian C. Smith

**Affiliations:** ^1^ Department of Biochemistry, Medical College of Wisconsin, Milwaukee, Wisconsin, USA, mcw.edu; ^2^ Structural Genomics Unit, Linda T. and John A. Mellowes Center for Genomic Sciences and Precision Medicine, Medical College of Wisconsin, Milwaukee, Wisconsin, USA, mcw.edu; ^3^ Department of Surgery, Medical College of Wisconsin, Milwaukee, Wisconsin, USA, mcw.edu; ^4^ Program in Chemical Biology, Medical College of Wisconsin, Milwaukee, Wisconsin, USA, mcw.edu

## Abstract

Polybromo‐1 (PBRM1) modulates chromatin accessibility and transcription via six bromodomains that bind acetyl‐lysine residues on nuclear proteins. PBRM1 variants exist in ~40% of clear cell renal cell carcinoma (ccRCC) cases. PBRM1 loss correlates with improved responses to antiangiogenics and immune checkpoint blockade, which are the standard of care for ccRCC therapy. Missense variants cluster within PBRM1 bromodomains and are present in 16% of ccRCC cases, with unknown impacts on therapeutic response. Since in‐depth biophysical and cellular testing of the hundreds of PBRM1 missense variants reported in ccRCC and other cancer types is intractable, computational approaches can provide actionable variant impact assessments. We employed an integrated structural genomics analysis at the levels of protein sequence (2D), structure (3D), and molecular dynamics (4D) to evaluate the effects of all reported ccRCC‐associated missense variants in the second and fourth bromodomains of PBRM1 (33 total) on molecular fitness (i.e., stability, structural integrity, and ligand binding). We also evaluated the concordance between molecular fitness and biophysical parameters of variant structural integrity (differential scanning fluorimetry, circular dichroism, and ^1^H‐NMR spectroscopy) and ligand binding (AlphaScreen and electrophoretic mobility shift assays). Integrated molecular fitness scores correlated with measures of acetylated histone binding, while 3D and integrated molecular fitness scores correlated with measures of thermal stability. This integrated structural genomics analysis accelerates mechanistic understanding of how ccRCC missense variants impact PBRM1 bromodomain structure and function. This knowledge supports future studies correlating specific missense variants with PBRM1 tumor‐suppressive functions and patient responses to antiangiogenics and immune checkpoint blockade to inform ccRCC personalized medicine approaches.

## 1. Introduction

Renal cell carcinoma is the deadliest genitourinary cancer, with a 12% 5‐year survival rate for advanced disease [1]. Clear cell renal cell carcinoma (ccRCC) is the most common subtype (~75% of cases) of renal cell carcinoma [2–4]. At the cellular level, ccRCC is characterized by disruption of epigenetic homeostasis [5, 6]. Members of the SWItching/sucrose nonfermentable (SWI/SNF) chromatin remodeling complex family are key players in this dysregulation; SWI/SNF proteins are mutated in ~50% of ccRCC cases [2]. Notably, the gene encoding the polybromo‐1 (PBRM1) protein, an eponymous component of the SWI/SNF polybromo, BRG1/BRM‐associated factor (PBAF) complex [7–10], is mutated in ~30%–40% of ccRCC cases [2, 11–13]. The PBAF complex binds to epigenetic histone modifications and regulates transcription by facilitating ATP‐dependent nucleosome organization and modulating DNA accessibility [10, 14, 15].

The chromatin‐binding functions of the PBAF complex are mediated in part by the six bromodomains of PBRM1 (BD1‐6) [9, 16, 17]. Bromodomains are ~110‐amino acid protein interaction modules that “read” epigenetically modified acetyl‐lysine (Kac) residues on histones [9, 17–19] and other nuclear proteins [20]. We and others have also reported that the PBRM1 bromodomains can bind nucleic acids [21, 22]. PBRM1 functions as a putative tumor suppressor in ccRCC and other cancer types [20, 23–25]. In this role, PBRM1 silences transcription around DNA double‐stranded breaks (DSBs) and promotes the repair of a subset of those DSBs [26]. PBRM1 also contributes to cell senescence by binding acetylated p53 [20]. Therefore, loss of PBRM1 tumor suppressor activities in ccRCC promotes cancer progression by disrupting the homeostatic relationship between the “histone language” of epigenetic chromatin modifications and PBAF‐mediated transcriptional regulation and DNA repair [9, 16, 17].

Intriguingly, loss‐of‐function variants that ablate PBRM1 protein expression in ccRCC are correlated with improved patient responses to antiangiogenic drugs [27–29] and immune checkpoint blockade (ICB) [30, 31], which, in combination, are the current standard of care for metastatic ccRCC [32, 33]. However, PBRM1 missense variants are present in ~16% of ccRCC cases [12]. Such variants result in the expression of full‐length PBRM1 protein missense variants whose impacts on PBRM1 protein structure, Kac binding, tumor suppressor activity, and response to ccRCC therapies are largely unknown [34]. The number of disease‐associated variants cataloged in the disease‐focused ClinVar database is estimated to increase by 60% each year [35]. Moreover, thousands of PBRM1 missense variants have been identified in ccRCC and other cancer types [12]. Therefore, determining the functional impacts of every cancer‐associated PBRM1 missense variant on patient responses to ccRCC therapies through in‐depth biophysical and cellular analyses is intractable [36]. Computational assessments of the impacts of missense variants on PBRM1 structure and function can identify potential mechanisms by which PBRM1 missense variants contribute to the pathobiology of ccRCC. Moreover, correlating computational and biophysical measurements can identify which computational metrics are useful in predicting patient responses to antiangiogenics and ICB and inform precision medicine approaches for ccRCC.

Numerous computational algorithms exist that use protein sequence as the primary input (2D) to predict the effects of residue‐level alterations on overall protein structure and function [37–39]. With these tools, variants are assigned a numerical score between 0 and 1 and annotated as “benign” (closer to 0), “variant of uncertain significance,” or “pathogenic” (closer to 1) vis‐à‐vis the anticipated impacts of the variant in a given disease [37–39]. However, many 2D impact prediction algorithms are limited by minimal structural information inputs [38–40] or by an inability to link predicted missense variant impacts to relevant biological mechanisms [41]. To address these limitations, our previous studies of other epigenetic proteins integrated 2D variant impact prediction tools into a multilevel structural genomics analysis [42–46]. The second bromodomain (BD2) and fourth bromodomain (BD4) are the two most important PBRM1 bromodomains for interacting with histone ligands and the tumor‐suppressive functions of full‐length PBRM1 [20, 23, 24, 47]. Therefore, we employed our integrated structural genomics analysis to computationally predict the impacts of all reported ccRCC PBRM1‐BD2 and ‐BD4 missense variants (33 total) on bromodomain stability and histone Kac peptide binding. Improving upon our previous structural genomics analyses that relied solely on computation [42–46], we validated our computational predictions by assessing the effects of PBRM1 bromodomain missense variants on bromodomain stability, structural integrity, and ligand binding in vitro. By integrating our computational and biophysical results to determine which computational metrics best correlate with experimental data, we provide the first reported annotation of PBRM1 bromodomain variant molecular fitness relative to the expected and actual variant impacts on stability, structural integrity, and ligand binding. The results of our study demonstrate that our integrated structural genomics analyses generate actionable hypotheses on the mechanisms by which PBRM1 variants contribute to ccRCC, supporting future studies correlating specific missense variants with patient responses to antiangiogenics and ICB, thereby accelerating ccRCC precision medicine initiatives.

## 2. Materials and Methods

### 2.1. PBRM1 Bromodomain Missense Variant Identification

PBRM1 bromodomain missense variant identification was performed as previously described [22]. Data comprising all known ccRCC–associated variants in the BD2 and BD4 of PBRM1 (ENST00000296302) identified by next‐generation sequencing and curated in the Catalogue of Somatic Mutations in Cancer (COSMIC; https://cancer.sanger.ac.uk/cosmic) [12] database were mined from COSMIC on October 2, 2025. The number of variants per variant type was determined by recording the total number of entries listed in the COSMIC positive data table for each variant type. The number of missense variants per PBRM1 functional domain was determined by downloading the COSMIC positive data table of PBRM1 missense variants and identifying those that fell within PBRM1 functional domains, with domain boundaries defined by InterPro SMART annotations (https://www.ebi.ac.uk/interpro/) [48]. Nine PBRM1‐BD2 missense variants and 10 PBRM1‐BD4 missense variants found in the general population with allele frequencies between ~6 × 10^−7^ and 4 × 10^−5^ were collected from the Genome Aggregation Database (gnomAD; https://gnomad.broadinstitute.org/) [49]. We previously conducted a biophysical assessment of seven PBRM1‐BD4 ccRCC missense variants (M523R, N528I, R576P, Y580C, M586T, N601K, and S605F) [22]. These variants were included in the current study to determine the concordance between computational and in vitro assessments of the impacts of missense variants on bromodomain structure and function.

### 2.2. Sequence‐Based (2D) Missense Variant Biochemical Fitness Data Collection and Classification

2D missense variant biochemical fitness data collection and classification were performed as previously described [45]. The Database for Nonsynonymous SNPs′ Functional Predictions (dbNSFP; http://database.liulab.science/dbNSFP) v. 4.7 [50–52] was used to annotate the missense variants with rank scores from 48 prediction algorithms. The genomic location and altered base data for each missense variant, derived from the GRCh38 reference assembly, were used as input to dbNSFP v. 4.7.

### 2.3. Molecular Modeling

Molecular modeling was performed as previously described [45]. The crystal structures of PBRM1‐BD2 (Protein Data Bank [PDB] ID 3HMF) [19], PBRM1‐BD4 (PDB ID 3TLP) [19], and an 11‐mer H3K14ac peptide (H3 Amino Acid Residues 9–19) (PDB ID 4QC1) [53] were retrieved from the RCSB PDB [54] and used as geometric references for constructing the biomolecular models. The H3K14ac peptide was truncated to 10 amino acid residues (H3 Amino Acid Residues 10–19), as only these residues were ordered in the original crystal structure. Missense variants were built by substitutions into the wild‐type (WT) (WT:PBRM1‐BD2 or WT:PBRM1‐BD4) protein; side chain refinement was performed on mutated residues with the ChiRotor algorithm [55]. Input conformations for MD simulations with the Discovery Studio suite Version 21.1 (Dassault Systèmes BIOVIA; https://www.3ds.com/products/biovia) were obtained as follows: Structures were first energy‐minimized using the Steepest Descent function over a 500 ps run at constant volume and 300 K, followed by 500 ps of a conjugate gradient minimization at constant volume and 300 K with the distance‐dependent dielectric solvent model.

### 2.4. Computational Mutagenesis and Energy Calculations

Computational mutagenesis and energy calculations were performed as previously described [45]. The effects of single‐point variants in PBRM1 bromodomain stability were evaluated by calculating the change in folding free energy (*Δ* *Δ* *G*_folding_) with two different force field‐based methods. The CHARMM force field was used to assess the impact of variants on structural integrity (*Δ* *Δ* *G*_fold−CHARMM_) using the calculate mutation energy (stability) protocol [56] in Discovery Studio. We also evaluated changes in bromodomain stability using FoldX [57] (*Δ* *Δ* *G*_fold−FOLDX_) for each missense variant. The effect of a variant on protein stability (*Δ* *Δ* *G*_folding_) is calculated as the difference between the Gibbs free energies of folding (*Δ* *G*_folding_) of the missense variant (*Δ* *G*_folding−missense variant_) and the WT bromodomain (*Δ* *G*_folding−WT_). The effects of single‐point variants on PBRM1 bromodomain binding to the H3K14ac ligand were evaluated with the calculate mutation energy (binding) protocol [58] in Discovery Studio. The difference between the Gibbs free energy of the bromodomain:ligand complex and the unbound bromodomain (*Δ* *Δ* *G*_binding_) is calculated as the difference between the binding free energy of the mutated bromodomain (*Δ* *G*_binding−mutant_) and the WT bromodomain (*Δ* *G*_binding−WT_). All energy terms were calculated by CHARMM with a generalized Born implicit solvent model. Missense variants were categorized as destabilizing (*ΔΔ* *G*_folding_ > 2 kcal/mol), neutral (*ΔΔ* *G*_folding_ between −2 and 2 kcal/mol), or stabilizing (*ΔΔ* *G*_folding_ < −2 kcal/mol).

### 2.5. Structural Perturbation Assessment

Global and local structure perturbations within PBRM1‐BD2 and ‐BD4 missense variants were assessed by measuring the positional displacement of backbone atoms (global) and only the atoms near the affected residue of interest (local). For global structure perturbation, the entire backbone atoms were used to calculate the root mean square deviation (RMSD) between the WT and missense variant structures. For local structure perturbation, residues within a 5 and 10 Å radius from the variant site were separately selected, and backbone atoms were used for calculating the least‐squares RMSD between the WT and missense variant structures with PyMOL v. 3.1.3 (Schrödinger, LLC).

### 2.6. Molecular Dynamics Simulations

MD simulations were performed as previously described [45]. MD simulations were performed with the CHARMM [59] all‐atom force field in Discovery Studio. Distance‐dependent dielectrics were used as an implicit solvent model with a dielectric constant of 80 at pH 7.4. Each model was initially subjected to 5000 steps of energy minimization with the Steepest Descent function, followed by 5000 steps of conjugate gradient without any constraints/restraints on the PBRM1‐BD2 or ‐BD4 atoms. The system was then heated to 300 K at a constant volume over 200 ps, followed by 500 ps of equilibration. Then, 10 ns production simulations were conducted under the isothermal‐isovolumetric (NVT) thermodynamic ensemble with a step size of 2 fs. Trajectory files were recorded every 0.01 ns simulation step to generate 1000 conformations. Trajectory files were analyzed for structural impacts. The RMSD was calculated by using the protein backbone as a reference. The root mean square fluctuation (RMSF) from the average structure in the trajectory was calculated by superimposing each frame onto the first frame and averaging the resulting structures. The radius of gyration (*R*_*g*_) and solvent‐accessible surface area (SASA) were also calculated using the tools available within Discovery Studio. All RMSD, RMSF, *R*_*g*_, and SASA values for PBRM1 missense variants were then subtracted from WT values for use as 3D and 4D variant impact metrics. The data were plotted in GraphPad Prism, and molecular visualizations were generated using PyMOL.

### 2.7. Molecular Mechanics–Poisson–Boltzmann Surface Area (MM‐PBSA) Calculations

MM‐PBSA calculations were performed as previously described [45] to evaluate the binding affinities of PBRM1‐BD2 or ‐BD4 interactions with the H3K14ac peptide [60, 61]. The last 1000 ps of the MD trajectories were analyzed at a 10 ps time interval to estimate the free energy of ligand binding (*Δ* *G*_bind_), which was calculated by the following equation:

(1) ΔGbind=Gcomplex−GPB1BD−Gligand,

where *G* ^complex^, *G* ^PB1BD^, and *G* ^ligand^ are the free energies of PBRM1‐BD2/BD4:H3K14ac complexes, PBRM1‐BD2/4 (WT and missense variants), and ligand (H3K14ac peptide), respectively. The free energy (*G*) of each state is estimated as follows:

(2) G=EMM+GPB+GSA−TS,

(3) EMM=Evdw+Eele+Ebnd,

where in Equation (2), *E*_MM_ is the molecular mechanical energy, *G*_PB_ and *G*_SA_ are the polar and nonpolar contributions to the solvation‐free energies, and TS is the entropic contribution of the system. *E*_MM_ was obtained by summing the contributions from van der Waals (*E*_vdw_), electrostatic (*E*_ele_), and bonded (*E*_bnd_; bond, angle, and dihedral) interactions, as described in Equation (3). Finally, the change in free energy of missense variant ligand binding relative to the WT bromodomains (*ΔΔ* *G*_MM−PBSA_) was calculated. We also calculated interaction energy (CIE) to evaluate nonbonded interactions (van der Waals and electrostatic terms) (*ΔΔ* *G*_CIE_) between the H3K14ac peptide and PBRM1‐BD2/4 (WT and missense variants) using tools in Discovery Studio.

### 2.8. Integrated Molecular Fitness Scoring and Variant Annotation

Impact scores from each level of analysis (2D, 3D, and 4D) were independently determined by converting the raw values from each analysis metric to *Z* scores using Equation (4).

(4) Z score=x−x¯S,

where *x* is the raw value from an analysis metric, $\bar{x}$ is the average of all raw values from an analysis metric, and *S* is the standard deviation of the sample. Next, the *Z* scores were converted to scaled *Z* scores from 0 to 1 using Equation (5).

(5) Scaled Z score=x−minxmaxx−minx,

where *x* is the *Z* score, min (*x*) is the minimum value of all *Z* scores for a given metric, and max (*x*) is the maximum value of all *Z* scores for a given metric. Finally, the scaled *Z* scores from each metric were combined and averaged with equal weights separately for PBRM1‐BD2 and ‐BD4 variants. Combined 3D/4D impact scores and integrated molecular fitness score (IMFS) were determined by combining and averaging the scaled *Z* scores from each metric with weights assigned by Equation (6).

(6) w=1−nlntot,

where *n*_l_ is the number of metrics in the relevant level of the integrated computational analysis and *n*_tot_ is the total number of metrics across all three levels of the integrated computational analysis. PBRM1 missense variants were assigned to one of three annotation categories based on IMFS (0–0.43: tolerated; 0.43–0.53: VUS; and 0.53–1: damaging). Damaging variants were also subclassified based on the difference between 3D and 4D scores (4D − 3D < −0.17: structural variant [SV]; 4D − 3D > 0.17: dynamic variant [DV]; and −0.17 < 4D − 3D < 0.17: structural/dynamic variant [SDV]). The optimal IMFS variant annotation threshold was determined by receiver operating characteristic (ROC) [62] curve analysis based on H3K14ac peptide binding (variant binding with normalized AlphaCounts ≥ 0.95 at 10 *μ*M protein) to maximize sensitivity and specificity according to Youden′s index [62]. Absolute uncertainty in the optimal IMFS threshold for variant annotation was calculated from all possible annotation thresholds within 0.12 absolute standard deviations of the optimal threshold. Damaging variant subclassification thresholds were determined by ROC based on H3K14ac peptide binding (variant binding with normalized AlphaCounts ≤ 0.01 at 10 *μ*M protein) to maximize sensitivity and specificity using Youden′s index [62]. All ROC analyses were conducted in GraphPad Prism, and 95% confidence intervals were determined with the Wilson/Brown method.

### 2.9. Principal Component Analysis (PCA)

Forty‐four 2D metrics, 5 3D metrics, and 2 4D metrics from the final integrated computational analysis as well as *Δ* *T*_*m*_, circular dichroism (CD) signal at 208 and 222 nm, average NMR peak intensity from 0 to 1.25 pm and 6.9 to 9.5 ppm, and normalized AlphaCounts (0.1, 1, 10, and 0.1–10 *μ*M) were used as input for PCA, which was performed in R using prcomp. Computational metrics were first scaled and weighted in R before PCA to ensure that 2D, 3D, and 4D analysis levels contributed equally to the PCA, using Equation (7).

(7) y=x−μσ×1m,

where *x* is the raw value from an analysis metric, *μ* is the mean of all raw values from an analysis metric, *σ* is the standard deviation of all raw values from an analysis metric, and *m* is the number of metrics comprising a given level of the structural genomics analysis. Insoluble missense variants without biophysical data were imputed “worst‐case” biophysical outcomes (*Δ* *T*_*m*_ derived by subtracting the melting curve initiation temperature [20°C] from the WT bromodomains; CD and NMR signal derived from variants N258I and L526S, which exhibited the greatest disruption of secondary and tertiary structure; normalized AlphaCounts of 0 relative to the WT bromodomains). PCA plots were generated in R and transferred to Adobe Illustrator for presentation. P550L was also imputed “worst‐case” *Δ* *T*_*m*_ derived by subtracting the melting curve initiation temperature (20°C) from the WT bromodomains.

### 2.10. Statistical Analysis

Correlation analyses to determine Pearson correlations were conducted in GraphPad Prism with two‐tailed *t*‐tests.

## 3. Results

### 3.1. ccRCC‐Associated Missense Variants Cluster in PBRM1 Bromodomains

**Figure 1:**
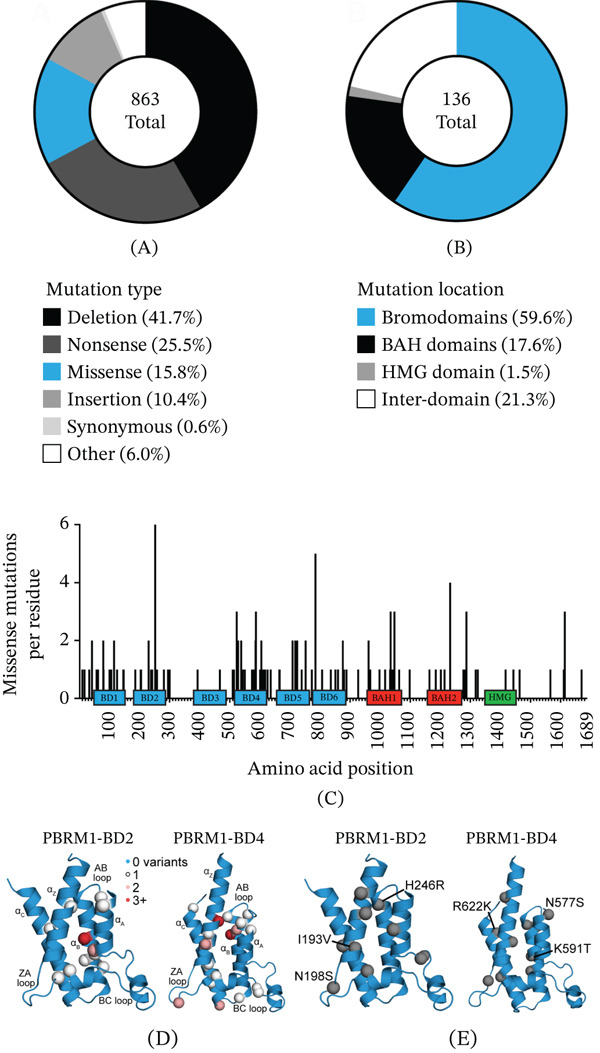
ccRCC‐associated PBRM1 missense variants are concentrated in PBRM1 bromodomains. (A) Percentage of ccRCC‐associated PBRM1 variants by variant type, as annotated in the Catalogue of Somatic Mutations in Cancer (COSMIC) [12] database. (B) Percentage of ccRCC‐associated PBRM1 missense variants by functional domain, as annotated in COSMIC. (C) ccRCC‐associated PBRM1 missense variants per residue across the entire protein sequence, as annotated in COSMIC. Bromodomains (BD; blue), bromo‐adjacent homology domains (BAH; red), and high‐mobility group (HMG; green) domains are denoted, with domain boundaries determined from InterPro [48] annotations. (D) ccRCC‐associated missense variants per residue in PBRM1‐BD2 (PDB ID 3HMF) [19] and PBRM1‐BD4 (PDB ID 3TLP); [19] impacted residues examined in this study are represented as spheres and color‐coded by the number of missense variants per residue identified in COSMIC. (E) PBRM1‐BD2 and ‐BD4 missense variants identified in the general population from the Genome Aggregation Database (gnomAD); impacted residues examined in this study are represented as dark gray spheres. Population variants selected for biophysical analysis in this study are labeled.

To interrogate the variant landscape of PBRM1 in ccRCC, we extracted all known ccRCC‐associated PBRM1 variants from the COSMIC database [12]. The most abundant types of PBRM1 genetic alterations are deletions and nonsense variants (collectively 67% of all PBRM1 variants in ccRCC) (Figure 1A), which result in the loss of full‐length PBRM1 protein. Loss of full‐length PBRM1 protein elevates angiogenic signaling in ccRCC [63, 64] and correlates with improved patient response to antiangiogenic [27–29] and ICB therapies [30, 31]. However, missense variants, which comprise 16% of PBRM1 variants in ccRCC (Figure 1A), generate full‐length protein variants whose function and contributions to ccRCC, as well as patient responses to ccRCC therapies, are unknown. We and others have shown that PBRM1 missense variants predominantly cluster (60%) in bromodomains (Figure 1B), the epigenetic histone Kac “reader” modules critical for PBRM1 function [22–24, 47]. By mapping ccRCC missense variants across the PBRM1 primary sequence, the BD2 and BD4 together comprise 13% of the entire protein sequence, yet contain 32% of ccRCC‐associated variants (Figure 1C). In contrast, the first, third, fifth, and sixth bromodomains collectively account for 26% of the entire protein sequence and contain 28% of ccRCC‐associated PBRM1 missense variants (Figure 1C). Therefore, PBRM1‐BD2 and ‐BD4 are missense variant hotspots relative to the other four PBRM1 bromodomains in ccRCC.

PBRM1‐BD2 and ‐BD4 are also the most important of the six PBRM1 bromodomains for interacting with modified histone peptides and nucleosomes in vitro and chromatin in cells [23, 24, 47]. PBRM1‐BD4 also interacts with acetylated p53 [20, 23, 24, 47]. Additionally, PBRM1‐BD2 and ‐BD4 are critical for the tumor‐suppressive functions of full‐length PBRM1, as mutations that disrupt the conserved Kac‐binding Asn residue in either bromodomain increase the proliferation of ccRCC cell lines in vitro [23, 47] and the growth of ccRCC tumors in mouse xenograft models [20, 24]. We therefore selected all known PBRM1‐BD2 (12 variants) and ‐BD4 (21 variants) missense variants associated with ccRCC to assess their impact on bromodomain stability and ligand binding function. These missense variants cluster in the ZA and BC loops, which comprise the histone Kac binding pocket [18, 19, 65], or in helical regions that facilitate bromodomain core helical packing [19] (Figure 1D). We also identified PBRM1‐BD2 (9 variants) and ‐BD4 (10 variants) missense variants with allele frequencies between 6 × 10^−7^ and 4 × 10^−5^ from the gnomAD [49, 66], which primarily contains genome sequencing data from individuals included as healthy controls in clinical trials [66]. Therefore, we considered these 19 PBRM1 variants as “population” missense variants and putative negative controls for our analyses (Figure 1E). Because the variants analyzed in this study are both proximal and distal to key structural and functional regions of the PBRM1 bromodomains (Figure 1E), we hypothesized that the ccRCC‐associated missense variants exhibit a range of impacts on bromodomain stability and ligand binding. In contrast, we anticipated that population missense variants would retain bromodomain stability and ligand binding properties comparable to those of WT bromodomains.

### 3.2. Integrated Structural Modeling Refines the Prediction of PBRM1 Variant Impacts Over Sequence‐Based Tools

**Figure 2:**
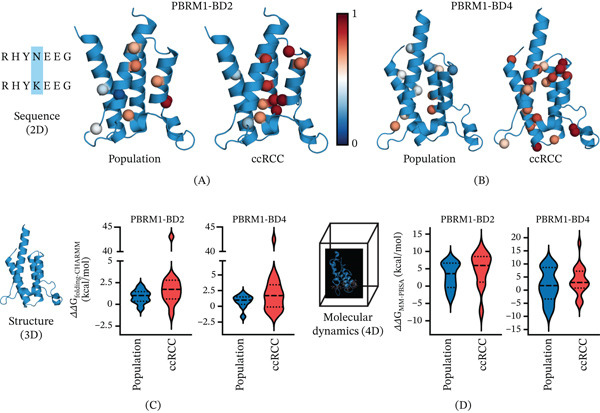
Integrating structural and dynamics‐based molecular fitness assessments with sequence‐based algorithms. (A) Population and ccRCC PBRM1‐BD2 missense variant 2D scores mapped onto structures. (B) Population and ccRCC PBRM1‐BD4 missense variant 2D scores mapped onto structures. (C) Predicted effects of population and ccRCC PBRM1‐BD2 and ‐BD4 missense variants on bromodomain stability relative to WT, as assessed by *ΔΔ* *G*_fold−CHARMM_. (D) Predicted effects of population and ccRCC PBRM1‐BD2 and ‐BD4 missense variants on bromodomain ligand binding relative to WT, as assessed by *ΔΔ* *G*_MM−PBSA_.

The effects of the 33 ccRCC and the 19 population missense variants on PBRM1‐BD2 and ‐BD4 stability and function were first probed with 48 sequence‐based (2D) variant impact prediction tools [37–39], including Polyphen2 [56], Protein Variation Effect Analyzer (PROVEAN) [67], Rare Exome Variant Ensemble Learner (REVEL) [68], and Combined Annotation Dependent Depletion (CADD) [69] (Figure 2A,B and Table S1). Professional clinical societies, such as the American Society of Clinical Oncology and the American College of Medical Genetics and Genomics, recommend several of these 2D tools [37, 38]. 2D impact tools typically assign missense variants a score between 0 and 1, where scores closer to 0 are predicted to be tolerated for protein structure and function. In contrast, scores closer to 1 are predicted to be damaging. The 2D impact tools predicted that 94% of the PBRM1 ccRCC variants and 53% of the PBRM1 population variants are damaging for bromodomain stability and function (2D impact score > 0.5) [70] (Figure 2A,B and Table S1). Therefore, 2D impact tools predict that both ccRCC and population PBRM1 missense variants broadly exhibit decreased stability and ligand binding relative to the WT PBRM1 bromodomains. However, the majority of 2D tools have been trained on similar datasets and statistical assumptions [37, 38, 71], resulting in similar predictions of the functional impacts of missense variants [38]. These biases in data sources and data processing may overestimate the number of PBRM1 ccRCC variants annotated as damaging for bromodomain stability and function [37, 38].

As we have previously done for other epigenetic proteins [42–46], we also probed ccRCC and population PBRM1 missense variants using a panel of structure‐based (3D) and molecular dynamics‐based (4D) metrics to predict the impacts of these variants on PBRM1 bromodomain stability and function through integrated structural genomics analyses. These predictions are informed by crystal structures obtained from the PDB (stressed) or energy‐minimized structures (relaxed) of PBRM1‐BD2 and ‐BD4. 3D impact assessments included calculations of the change in Gibbs free energy of missense variant folding relative to the WT protein (*ΔΔ* *G*_folding_) and structural perturbations as a proxy for protein stability [72] (Figure 2C and Table S2). Using *ΔΔ* *G*_folding_ with CHARMM (*ΔΔ* *G*_folding−CHARMM_), we found that PBRM1‐BD2 and ‐BD4 ccRCC variants trended towards greater instability relative to the WT bromodomains (positive *ΔΔ* *G*_folding_) (Figure 2C and Table S2). In contrast, PBRM1‐BD2 and ‐BD4 population variants trended towards equivalent or increased stability relative to the WT bromodomains (0 or negative *ΔΔ* *G*_folding_) (Figure 2C and Table S2). In contrast to the 2D impact predictions, 3D impact prediction tools suggest that population PBRM1 missense variants generally maintain stability relative to WT bromodomains, while ccRCC PBRM1 missense variants generally lose stability relative to WT bromodomains. By operating independently of the datasets and statistical assumptions used to train 2D impact tools [37, 38, 71], we anticipate that 3D impact tools generate a more accurate distribution of missense variant functional impact predictions than 2D tools. However, 3D impact tools alone are insufficient to fully distinguish population variants from ccRCC variants.

4D impact assessments included the change in Gibbs free energy of missense variant binding to acetylated histone ligands relative to the WT protein (*ΔΔ* *G*) [73] (Figure 2D and Table S2) and measures of dynamic protein stability [45, 74] (e.g., SASA [75] and radius of gyration (*R*_*g*_) [76]) (Table S2). PBRM1 bromodomains bridge the histone language and PBAF‐mediated transcriptional regulation [9, 16–18] by acting as epigenetic “readers” of H3K14ac [17, 23, 24, 47]. Using *ΔΔ* *G* with the MM‐PBSA technique (*ΔΔ* *G*_MM−PBSA_), PBRM1‐BD2 and ‐BD4 population and ccRCC variants exhibit a wide range of stabilizing (negative *ΔΔ* *G*_MM−PBSA_), neutral (*ΔΔ* *G*_MM−PBSA_ = 0), and destabilizing (positive *ΔΔ* *G*_MM−PBSA_) predicted impacts on H3K14ac peptide binding relative to WT PBRM1‐BD2 and ‐BD4 (Figure 2D and Table S2). Taken together, 4D impact prediction tools suggest that PBRM1‐BD2 and ‐BD4 missense variants exhibit a broader range of ligand binding capacity than 2D impact tools indicate. As with the 3D impact tools, we anticipate that 4D impact tools also generate a more accurate distribution of missense variant functional impact predictions than 2D tools [37, 38, 71], yet they alone are insufficient to fully distinguish population variants from ccRCC variants.

### 3.3. PBRM1 ccRCC Variants Compromise Bromodomain Thermal Stability

To validate the predictions of ccRCC missense variant impacts on PBRM1‐BD2 and ‐BD4 structure and function, we expressed all 12 PBRM1‐BD2 and 21 PBRM1‐BD4 ccRCC missense variants studied computationally (33 total) as individual constructs for analysis of their structural integrity and ligand binding capacity in vitro (Supporting Materials and Methods). We previously evaluated the structural stability and ligand binding of seven of the 21 PBRM1‐BD4 ccRCC missense variants [22]. We also previously validated PBRM1‐BD4 N601A as a functional control that disrupts PBRM1‐BD4 histone Kac binding, since the amide nitrogen of the conserved N601 sidechain is required to form a hydrogen bond with the Kac carbonyl oxygen in the bromodomain acetyl‐lysine binding pocket [19, 65]. For this study, we correspondingly generated PBRM1‐BD2 N263A as a functional control to disrupt PBRM1‐BD2 Kac binding. We also selected three PBRM1‐BD2 (I193V, N198S, and H246R) and three PBRM1‐BD4 (N577S, K591T, and R622K) population variants (Figure 1E) dispersed across diverse bromodomain regions with average scores < 0.4 across all 14 3D/4D impact metrics (Table S2) for purification and testing as predicted negative controls.

Despite multiple attempts, we were unable to purify seven PBRM1‐BD2 ccRCC missense variants (L186R, L230R, Y242H, I252R, I252T, L255H, and A256P) and one PBRM1‐BD4 missense variant (M586K) due to low protein solubility in vitro. L186 and L230 are conserved hydrophobic residues in the α_Z_ and α_A_ helices, respectively, that orient towards the bromodomain helical core to stabilize the core [19]. Y242 is a highly conserved residue in the AB loop that forms a hydrogen bond with a conserved Asp residue in the neighboring α_B_ helix. L252, L255, A256, and M586 are hydrophobic residues in the conserved α_B_ helix sequence *ϕ*xxD*ϕ*xx*ϕϕ*xN*ϕ*xxY/F, where *ϕ* is a hydrophobic amino acid and x is any amino acid [19]. Therefore, PBRM1 bromodomain solubility is highly sensitive to the disruption of residues that are critical for bromodomain stability.

**Figure 3:**
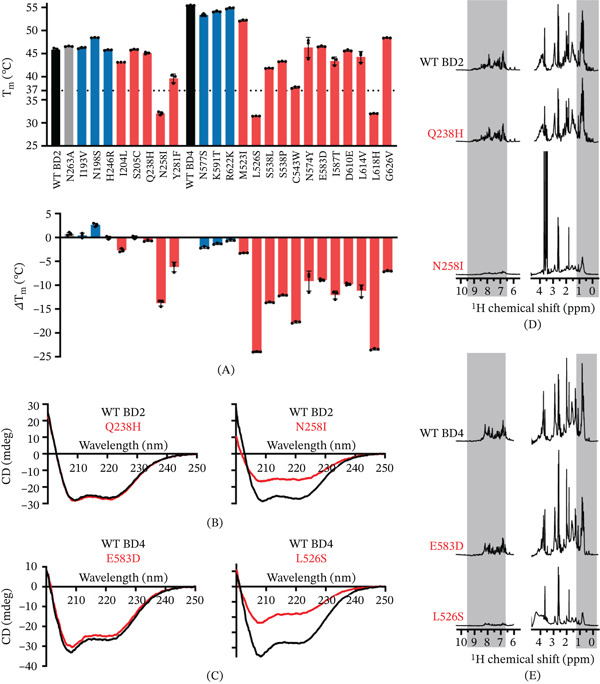
ccRCC‐associated PBRM1 bromodomain missense variants display decreased protein stability with intact secondary and tertiary structure integrity. (A) PBRM1‐BD2 and ‐BD4 population and ccRCC missense variant *T*_*m*_ determined by nanodifferential scanning fluorimetry (nanoDSF). WT shown in black, engineered functional controls in gray, population variants in blue, and ccRCC missense variants in red. The *Δ* *T*_*m*_ of variants relative to WT is also shown. Error bars represent SD; *n* = 3 for all constructs. (B) CD spectra of PBRM1‐BD2 ccRCC missense variants Q238H and N258I compared to PBRM1‐BD2 WT. (C) CD spectra of PBRM1‐BD4 ccRCC missense variants L526S and E583D compared to PBRM1‐BD4 WT. (D) ^1^H‐NMR spectra of PBRM1‐BD2 WT and ccRCC missense variants PBRM1‐BD2 ccRCC Q238H and N258I. Gray‐highlighted regions correspond to the spectral regions (backbone amide proton 6.5–9.5 ppm; saturated alkane methyl proton 0–1.25 ppm) used to assess variant tertiary structural integrity [22, 79, 80]. (E) ^1^H‐NMR spectra of PBRM1‐BD4 WT and ccRCC missense variants PBRM1‐BD4 L526S and E583D. The gray‐highlighted regions are as shown in (D).

For the 18 ccRCC‐associated variants, six population variants, and the functional controls we were able to purify, we employed nanodifferential scanning fluorimetry (nanoDSF) to determine the melting temperature (*T*_*m*_), an established measure of in vitro protein stability (Supporting Materials and Methods) [77, 78]. As anticipated, the six PBRM1‐BD2 and ‐BD4 population variants and the PBRM1‐BD2 functional control (N263A) [19, 65] retained thermal stability comparable to the WT bromodomains (Figure 3A and Table S3). Three of the five PBRM1‐BD2 ccRCC variants that we were able to express (I204L, S205C, and Q238H) exhibited minimal thermal destabilization, while PBRM1‐BD2 ccRCC variants N258I (*T*_*m*_32.1^°^C ± 0.4^°^C; *Δ* *T*_*m*_−13.7^°^C ± 0.6^°^C) and Y281F (*T*_*m*_39.7^°^C ± 1.0^°^C; *Δ* *T*_*m*_−6.2^°^C ± 1.0^°^C) were destabilized relative to WT PBRM1‐BD2 and the three PBRM1‐BD2 population variants (I193V, N198S, and H246R) (*T*_*m*_43.1^°^C–47.1^°^C) (Figure 3A and Table S3). Most of the 14 PBRM1‐BD4 ccRCC variants that were successfully purified exhibited substantial thermal destabilization (*T*_*m*_ 31.5^°^C–48.4^°^C; average *Δ* *T*_*m*_−11.8^°^C) relative to WT PBRM1‐BD4 and the three PBRM1‐BD4 population variants (N577S, K591T, and R622K) (*T*_*m*_ 53.4^°^C–55.5^°^C) (Figure 3A and Table S3). We were unable to determine a *T*_*m*_ value for the PBRM1‐BD4 ccRCC missense variant P550L (Table S3), suggesting that this variant exhibits high structural destabilization and is unfolded even at 20°C. Notably, four variants had *T*_*m*_ values below (N258I, L526S, and L618H) or within 2°C (C543W) of human body temperature (37°C) (Figure 3A and Table S3). Accordingly, several PBRM1 bromodomain ccRCC variants are likely to be at least partially unfolded under physiological conditions.

We also used CD to assess the impacts of PBRM1‐BD2 and ‐BD4 ccRCC missense variants on secondary structure integrity (Supporting Materials and Methods) [81, 82]. Since PBRM1 bromodomain secondary structure is primarily α‐helical (Figure 1D,E), folded bromodomains should exhibit characteristic α‐helix spectral minima at 208 and 222 nm [81, 82]. The CD spectra demonstrated that all six PBRM1 population variants and nearly all PBRM1‐BD2 and ‐BD4 ccRCC variants retained their secondary structure relative to the WT bromodomains, with Q238H and E583D representative of these results (Figure 3B,C and Figure S1) [22]. In agreement with the high structural destabilization observed by nanoDSF (Figure 3A and Table S3), PBRM1 ccRCC variants N258I and L526S exhibited decreased α‐helical content consistent with disrupted secondary structure integrity (Figure 3B,C). Overall, the retention of secondary structure across most variants tested indicates that the effects of most ccRCC‐associated missense variants on PBRM1 bromodomain stability and structural integrity are more local than global [22, 72].

To determine the effects of PBRM1‐BD2 and ‐BD4 ccRCC missense variants on tertiary structure integrity, we employed one‐dimensional proton NMR spectroscopy (^1^H‐NMR) (Supporting Materials and Methods). In ^1^H‐NMR experiments, distinct and well‐dispersed signals in the backbone amide proton (6.5–9.5 ppm) and saturated alkane methyl proton (0–1.25 ppm) regions are indicative of a well‐folded protein [22, 79, 80]. Only missense variants N258I and L526S exhibited a loss of ^1^H‐NMR signal in these key spectral regions, consistent with spectral broadening upon tertiary structure unfolding and/or protein aggregation (Figure 3D,E). However, all six PBRM1 population variants and the majority of PBRM1 ccRCC variants retained intact tertiary structure (Figure 3D,E and Figure S2), corroborating the largely intact PBRM1 ccRCC variant secondary structure observed by CD (Figure 3B,C and Figure S1) and the variable local impacts on bromodomain thermal stability observed by nanoDSF (Figure 3A and Table S3).

### 3.4. PBRM1 ccRCC Variants Exhibit Variable Defects in Histone Acetyl‐Lysine Recognition

**Figure 4:**
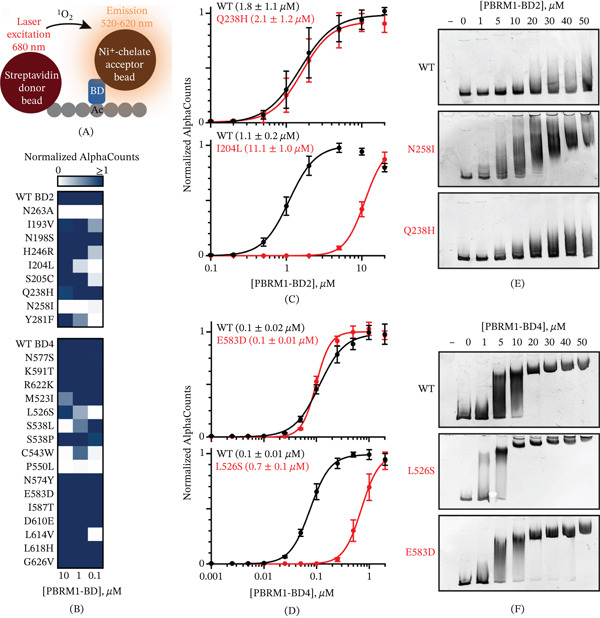
ccRCC‐associated PBRM1 bromodomain missense variants exhibit variable ligand binding capacity. (A) AlphaScreen assay schematic. (B) Heat map of PBRM1‐BD2 and ‐BD4 population and ccRCC missense variants (0.1–10 *μ*M) binding to a biotinylated H3K14ac peptide (100 nM), normalized to WT in AlphaScreen assays (*n* = 3). (C) AlphaScreen titrations of ccRCC missense variants PBRM1‐BD2 I204L and Q238H (0.1–20 *μ*M) binding to a biotinylated H3K14ac peptide (100 nM) (*n* = 3), where error bars represent SD. (D) AlphaScreen titrations of ccRCC missense variants PBRM1‐BD4 L526S and E583D (0.001–2 *μ*M) binding to a biotinylated H3K14ac peptide (100 nM) (*n* = 3), where error bars represent SD. (E) EMSAs of PBRM1‐BD2 WT and ccRCC missense variants Q238H and N258I (0–50 *μ*M) binding to 150 nM Widom 601 DNA (representative of *n* = 2). WT shown in black; ccRCC missense variants in light gray. (F) EMSAs of PBRM1‐BD4 WT and ccRCC missense variants L526S and E583D (0–50 *μ*M) binding to 150 nM Widom 601 DNA (representative of *n* = 2). WT shown in black; ccRCC missense variants in light gray.

Therefore, we assessed the functional impacts of PBRM1 ccRCC variants on binding to a H3K14ac peptide using AlphaScreen (Alpha = Amplified Luminescent Proximity Assay) (Figure 4A, Supporting Materials and Methods) [22, 83–85]. PBRM1‐BD2 and ‐BD4 population and ccRCC missense variants were screened for their ability to bind to an H3K14ac peptide relative to the WT bromodomains (Figure 4B and Table S4). As anticipated, all six PBRM1 population variants retained H3K14ac peptide binding (Figure 4B and Table S4) relative to the WT bromodomains. Also, as anticipated [19, 23, 65], the PBRM1‐BD2 control (N263A) demonstrated a complete loss of H3K14ac peptide binding (Figure 4B and Table S4). We previously reported a similar complete loss of H3K14ac peptide binding for the PBRM1‐BD4 N601A control [22].

In agreement with the structural destabilization observed by nanoDSF (Figure 3A and Table S3), ccRCC variants N258I and P550L exhibited a complete loss of H3K14ac peptide binding. We previously reported that seven of the PBRM1‐BD4 ccRCC variants (M523R, N528I, R576P, Y580C, M586T, N601K, and S605F) also disrupt H3K14ac peptide binding [22]. Surprisingly, the majority of the PBRM1 ccRCC variants in this study exhibited some degree of H3K14ac peptide binding (defined as AlphaScreen counts above background at 1 or 10 *μ*M PBRM1‐BD2 or ‐BD4 protein) in the initial AlphaScreen assays (Figure 4B and Table S4). To determine the relative H3K14ac peptide binding capacity of variants that retained the ability to bind the H3K14ac peptide, we performed full titrations of these variants. We compared these titrations with those of the WT bromodomains (Figure 4C,D, Figure S3, and Table S6). Of the 16 PBRM1 ccRCC variants subjected to full AlphaScreen titrations, eight variants (S205C, Q238H, S538P, E583D, I587T, L614V, L618H, and G626V) exhibited a half‐maximal effective concentration (EC_50_) of H3K14ac peptide binding within the standard error of the WT bromodomains (Figure 4C,D, Figure S3, and Table S6). All six population variants also had similar EC_50_ values of H3K14ac peptide binding relative to the WT bromodomains (Figure S3 and Table S6). The EC_50_ values of the other eight PBRM1 ccRCC variants (I204L, Y281F, M523I, L526S, S538L, C543W, N574Y, and D610E) were weakened by ~2–10‐fold (Figure 4C,D, Figure S3, and Table S6). Together, the results of these and our previous [22] Kac binding studies suggest that 36% of ccRCC missense variants (25 variants analyzed in total) completely disrupt H3K14ac peptide binding, 32% exhibit decreased H3K14ac peptide binding, and 32% retain peptide binding comparable to that of the WT. Therefore, the impacts of ccRCC missense variants on PBRM1 bromodomain ligand binding vary widely.

Consistent with the prediction that one‐third of all bromodomains are capable of nucleic acid binding [86–89], we and others have demonstrated that PBRM1 bromodomains bind nucleic acids at a site that partially overlaps the canonical Kac binding site [21, 22]. Therefore, we used electrophoretic mobility shift assays (EMSAs) to probe the functional impacts of PBRM1 ccRCC variants on binding to Widom 601 DNA, a classical nucleosomal positioning DNA sequence (Supporting Materials and Methods) [90]. We screened the PBRM1‐BD2 and ‐BD4 ccRCC variants for their ability to bind Widom 601 DNA relative to the WT bromodomains and validated that the His_6_ tag did not induce nucleic acid binding (Figure S4). All PBRM1 ccRCC and population variants possessed a nucleic acid binding capacity equal to or greater than that of the WT bromodomains (Figure 4E,F and Figure S4). Accordingly, PBRM1 bromodomain binding to nucleic acids is dependent on an array of nonspecific electrostatic interactions that are minimally disrupted by missense variants where only one amino acid residue is altered (Figure S5).

### 3.5. IMFSs Corroborate With Biophysical Data to Stratify Variant Impact Mechanisms

Since all PBRM1 missense variants, except the six population controls and two functional controls analyzed in this study, are found in patients with confirmed ccRCC, annotating PBRM1 ccRCC missense variants as benign, VUS, or pathogenic in ccRCC and other known diseases can be clinically misleading. Therefore, we redefined our missense variant impact annotation categories as tolerated, VUS, or damaging to directly describe the predicted and validated impacts of missense variants on PBRM1 bromodomain structural integrity and histone peptide binding [91]. To normalize the computational data and match the scale used by many 2D variant impact algorithms [37–39], we first converted the results of the 3D and 4D metrics to *Z* scores to ensure they followed a normal distribution [92] and then converted the *Z* scores to a 0–1 scale [44]. We then assessed the correlation between our biophysical and computational assessments of PBRM1 missense variant impacts (Tables S1–S4 and Figure S6). Scores from most 2D and 3D metrics were correlated with PBRM1 bromodomain stability and ligand binding in vitro (*r* = −0.10 to −0.70) (Figure S6). Of the 4D metrics, only *ΔΔ* *G*_MM−PBSA_ and *ΔΔ* *G*_CIE_ showed correlations with PBRM1 bromodomain stability and ligand binding (*r* = −0.10 to −0.33) (Figure S6).

**Figure 5:**
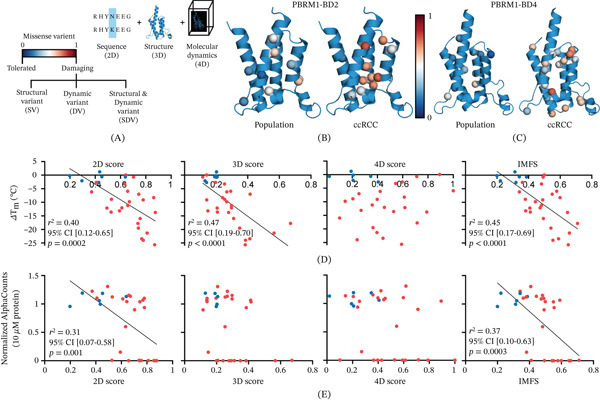
Structural and dynamic inputs improve clustering of PBRM1 missense variants. (A) Schematic of the integrated molecular fitness scoring pipeline. (B) Population and ccRCC PBRM1‐BD2 missense variants; mutated residues examined in this study are represented as spheres and gradient colored by integrated molecular fitness score (IMFS) (dark blue = 0 and dark red = 1). (C) Population and ccRCC PBRM1‐BD4 missense variants; mutated residues examined in this study are represented as spheres and gradient colored by integrated molecular fitness score (IMFS) (dark blue = 0 and dark red = 1). (D) Correlation between structural genomics pipeline scores and the stability of PBRM1‐BD2 and ‐BD4 missense variants, as assessed by nanoDSF (*Δ* *T*_*m*_). Population variants are shown in blue; ccRCC missense variants in red. (E) Correlation between pipeline scores and PBRM1‐BD2 and ‐BD4 missense variant ligand binding as assessed by the normalized AlphaScreen signal (10 *μ*M protein). Population variants shown in blue; ccRCC missense variants in red. Correlations are not presented for graphs where *r* ^2^ < 0.2.

We next combined the scaled *Z* scores for all metrics from the 2D, 3D, and 4D levels of our integrated structural genomics analyses, which correlate with the biophysical assessments of bromodomain stability and H3K14ac ligand binding (*r* < −0.2 for at least two of the five biophysical metrics), to calculate average scores for each level of the structural genomics analysis (2D, 3D, 4D). 2D, 3D, and 4D scores were combined with weights based on the total number of metrics per analysis level to generate IMFSs between 0 and 1 for PBRM1 missense variant annotation across diverse data inputs (Figure 5A–C), as we have previously done for other epigenetic proteins [42–46]. We then assessed the correlations between IMFS, individual 2D, 3D, and 4D impact scores, as well as combined 3D/4D impact scores, and biophysical assessments of missense variant thermal stability and H3K14ac peptide binding (Figure 5D,E). 2D scores correlated (*r* ^2^ = 0.40) with PBRM1 variant thermal stability (*Δ* *T*_*m*_ values), but 3D impact scores (*r* ^2^ = 0.47) and IMFS (*r* ^2^ = 0.45) correlated better with thermal stability (Figure 5D). Similarly, 2D scores correlated (*r* ^2^ = 0.31) with PBRM1 variant H3K14ac peptide binding (normalized AlphaCounts with 10 *μ*M of protein), but IMFS (*r* ^2^ = 0.37) exhibited a higher correlation with H3K14ac peptide binding (Figure 5E).

**Figure 6:**
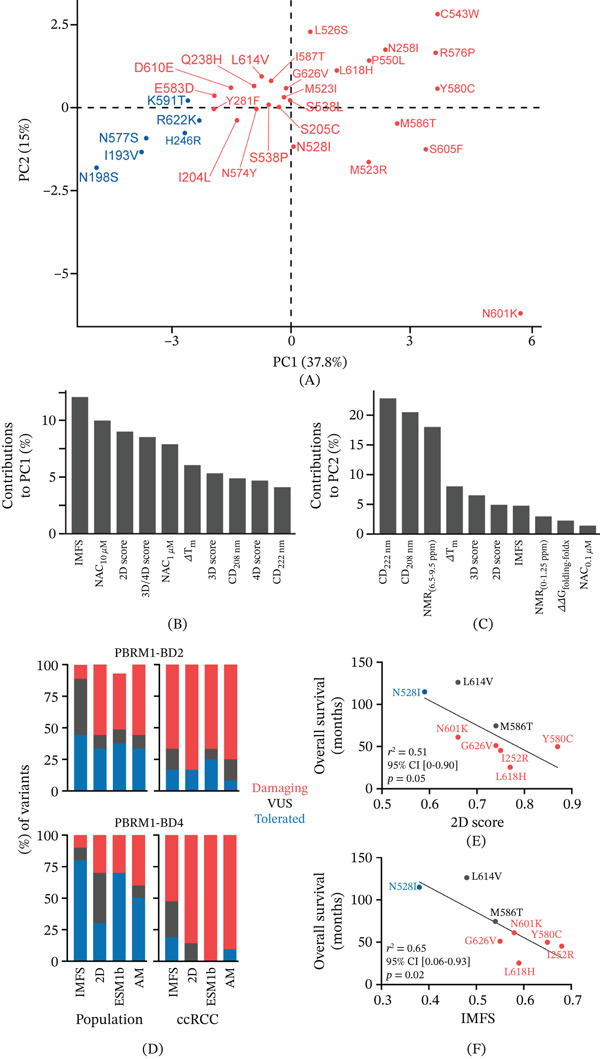
Computational and in vitro inputs inform PBRM1 missense variant impact annotations. (A) PCA of soluble PBRM1‐BD2 and ‐BD4 variants with computational and biophysical data from the integrated structural genomics analysis as inputs for variant clustering. (B) Contribution of the Top 10 metrics to the first principal component of the PCA plot. (C) Contribution of the Top 10 metrics to the second principal component of the PCA plot. (D) Population and ccRCC PBRM1‐BD2 and ‐BD4 missense variant annotations by scoring algorithm (AM = AlphaMissense). (E) Correlation between 2D and the overall survival of patients with ccRCC. Tolerated variants are shown in blue, VUS in gray, and ccRCC variants in red. (F) Correlation between IMFS and the overall survival of patients with ccRCC. Tolerated variants are shown in blue, VUS in gray, and ccRCC variants in red.

We also evaluated how the integrated structural genomics analysis identifies variants with similar computational and biophysical characteristics using PCA. PCA demonstrated that population and ccRCC variants cluster into distinct groups based on specific computational and ligand binding profiles (Figure 6A and Table S5). Notably, IMFS provided the greatest contributions to the first principal component, followed by PBRM1 variant H3K14ac peptide binding (normalized AlphaCounts with 10 *μ*M of protein) and 3D/4D scores (Figure 6B and Table S5). Secondary structure integrity (CD signal at 208 and 22 nm) and tertiary structure integrity (average NMR peak signal from 6.5 to 9.6 ppm) provided the greatest contributions to the second principal component (Figure 6C and Table S5). To ensure that the PCA was not biased by the absence of the eight ccRCC variants that we were unable to purify (L186R, L230R, Y242H, I252R, I252T, L255H, A256P, and M586K), we imputed “worst‐case” biophysical parameters to these variants for PCA. We observed that population and ccRCC variants remain clustered separately according to IMFS (Figure S7). Therefore, IMFSs have high utility for distinguishing PBRM1 ccRCC variants from population variants in the context of mechanistic variant interpretation.

To optimize the use of IMFS to annotate PBRM1 missense variants as tolerated, VUS, or damaging relative to structural integrity and histone peptide binding, we employed a ROC curve analysis [62] (Figure S8A and Table S6) based on H3K14ac peptide binding (variant binding with normalized AlphaCounts ≥ 0.95), the biophysical metric with the greatest contribution to the PBRM1 variant PCA (Figure 6C). The ROC curve analysis yielded an area under the curve of 0.87 (Figure S8A and Table S7), demonstrating that IMFSs informed by H3K14ac peptide binding have reliable predictive power. Based on this analysis, we assigned the 33 ccRCC and the 19 population PBRM1‐BD2 and ‐BD4 missense variants to three annotation categories according to the optimal IMFS threshold of 0.48 ± 0.05 for variant impact prediction (0–0.43: tolerated; 0.43–0.53: VUS; and 0.53–1: damaging) (Figure 6D and Table S8). In this ROC analysis, the optimal IMFS threshold for variant annotation was selected to maximize sensitivity and specificity, as indicated by Youden′s index [62]. The highest Youden′s index from the ROC analyses optimized the detection of true positives and true negatives [62], allowing robust estimation of the biological significance of any PBRM1 bromodomain missense variant with respect to its impacts on stability, structural integrity, and ligand binding in vitro.

We also separately considered the 3D and 4D impact scores (Table S9) of “damaging” PBRM1‐BD2 and ‐BD4 ccRCC variants to identify the putative mechanism of damage to the PBRM1 bromodomains [44]. We performed an ROC analysis based on the absolute difference between 3D and 4D scores and H3K14ac peptide binding (variant binding with normalized AlphaCounts ≥ 0.01) for all soluble damaging variants, again employing Youden′s index [62] to maximize sensitivity, specificity [62], and biological significance. The optimal 3D/4D threshold was 0.17 (Figure S8B and Table S9). Following this analysis, when 3D impact scores were higher than 4D impact scores (difference between scores > 0.17), “damaging” variants were further characterized as SVs, where the mechanism of damage is expected to be primarily mediated by disruption of PBRM1 bromodomain stability and structural integrity. When the difference between 3D and 4D impact scores is reversed, “damaging” variants were further categorized as DVs, where the mechanism of damage is anticipated to be primarily mediated by disruption of PBRM1 bromodomain acetylated histone binding. When 3D and 4D impact scores were similar (difference between scores < 0.17), “damaging” missense variants were further classified as SDVs, where the mechanism of damage is hypothesized to be mediated by disruption of both PBRM1 bromodomain stability and ligand binding.

Of the 19 “damaging” PBRM1‐BD2 and ‐BD4 ccRCC variants, Y242H, L255H, R576P, M586K, and G626V were classified as SVs; L230R, N258I, P550L, Y580C, N601K, and S605F were classified as DVs; and L186R, I252R, I252T, A256P, L526S, C543W, M586T, and L618H were classified as SDVs (Table S8). In agreement with the predicted SV and SDV mechanisms of disruption to PBRM1 bromodomain stability, we were unable to recombinantly purify three SVs (Y242H, L255H, and M586K) and four SDVs (L186R, I252R, I252T, and A256P) due to low solubility. Additionally, the secondary and tertiary structures of L526S (SDV) and R576P (SV) were compromised (Figure 4C,E) [22]. Consistent with the estimated mechanism of DV and SDV disruption to PBRM1 bromodomain ligand binding, N258I and P550L (DVs) exhibit a complete loss of H3K14ac peptide binding (Figure 4B and Table S4). We previously reported that Y580C, M586T, N601K, and S605F (DVs) are also unable to bind the H3K14ac peptide [22]. Additionally, L526S (SDV) and C543W (SDV) bound H3K14ac peptides with EC_50_ values 7‐ and 4‐fold weaker than WT PBRM1‐BD4, respectively (Figure S3 and Table S6). Unexpectedly, L618H (SDV) bound H3K14ac peptides with EC_50_ values only 2‐fold weaker than WT PBRM1‐BD4 (Figure S3 and Table S6), despite decreased thermal stability (*Δ* *T*_*m*_ −23.4°C) (Figure 3A and Table S3). Taken together, our studies provide critical structural and functional information that facilitate more comprehensive annotations of PBRM1 missense variants and mechanistic rationales for their effects in ccRCC, supporting future studies correlating specific missense variants with patient responses to ccRCC therapies.

## 4. Discussion

In this study, we integrated existing 2D missense variant impact prediction tools into a multitiered structural genomics analysis [42–46] with 3D and 4D predictions of missense variant impacts on protein structure and function. We applied this structural genomics analysis to identify the mechanisms by which PBRM1‐BD2 and ‐BD4 missense variants exhibit altered structure and/or function in the context of ccRCC, with implications for the role of PBRM1 missense variants in ccRCC pathobiology [34]. We also analyzed PBRM1 missense variants found in the general population with no disease associations as likely negative controls for our computational and biophysical studies. We determined the correlations between our computational studies (Figure 2) and biophysical assessments of the structural integrity (Figure 3) and ligand binding (Figure 4) of select PBRM1 missense variants. This allowed us to annotate PBRM1 ccRCC missense variants as “tolerated,” “VUS,” or “damaging.” We also further classified damaging variants as SVs, DVs, or SDVs based on their predicted and actual impacts on PBRM1 bromodomain structure and function, which cannot be interrogated from 2D analyses alone.

Biophysical assessments of bromodomain structural integrity and ligand binding generally agreed with the estimated SV, DV, and SDV mechanisms of disruption to PBRM1 bromodomain stability and ligand binding (Figures 3 and 4 and Table S8). However, we noted some discrepancies in variant annotation and the classification of “damaging” mechanisms. For example, Q238H was annotated as a “VUS” (IMFS = 0.49) (Table S8), yet exhibited minimal thermal destabilization (Figure 3A and Table S8) and bound to H3K14ac peptides (EC_50_ 2.1 *μ*M) just as well as WT PBRM1‐BD2 (EC_50_ 1.8 *μ*M). Here, 2D scores disproportionately elevated the IMFS, masking tolerated effects that became evident only when 3D and 4D metrics were considered together (Table S8). Therefore, for select VUS, structure‐ and dynamics‐based evaluations may be more accurate than sequence‐based tools in annotating variant impacts. Additionally, L230R, a “damaging” variant classified as a DV, could not be purified, consistent with disruption to bromodomain stability more characteristic of SVs or SDVs. Therefore, disrupted ligand binding may, in some cases, prioritize variants for degradation [93, 94]. Additionally, L618H, a “damaging” SDV variant, retained H3K14ac binding despite exhibiting structural destabilization (Figure 3). Accordingly, destabilization at distal residues may be insufficient to propagate into the binding pocket to affect histone Kac interactions in some cases [22, 72]. These discrepancies suggest that additional 3D and 4D impact metrics should be identified in future studies to improve the predictive performance of our integrated structural genomics analysis and reduce misannotation and misinterpretation of missense variants.

We compared our IMFS‐based PBRM1 variant annotations with variant annotations derived from 2D impact scores alone. We also compared our IMFS‐based annotations with variant annotations derived from large language models AlphaMissense [95] and ESM1b [96, 97], which are both 2D algorithms that use protein sequence as the primary input, but also incorporate structural information into variant impact analysis [95–97]. The same score thresholds used for IMFS annotation (0–0.43: tolerated; 0.43–0.53: VUS; and 0.53–1: damaging) were applied to PBRM1 variant annotations based on 2D, AlphaMissense, and ESM1b scores. Consistent with our hypothesis that population variants are negative controls for our ccRCC variant assessments, IMFS scores permitted annotation of 63% of the PBRM1‐BD2 and ‐BD4 population variants as tolerated—a clear improvement over the 2D impact (32%), ESM1b (53%), and AlphaMissense (42%) annotations (Figure 6). IMFS annotations agree well with our biophysical studies, where all six PBRM1 population variants retained structural integrity and ligand binding relative to the WT bromodomains (Figure 3 and Figures S1–S4). Although all four scoring systems yielded similar annotations of PBRM1‐BD2 ccRCC variants, IMFS scores permitted annotation of 19% of PBRM1‐BD4 ccRCC variants as tolerated and 52% as damaging for bromodomain structure and function. These results starkly contrast with the 2D, AlphaMissense, and ESM1b annotations, where few PBRM1‐BD4 ccRCC variants (0%–9.5%) are annotated as tolerated, and most variants (87.5%–100%) are annotated as damaging. In light of this comparison, IMFS annotations align with our AlphaScreen data, where 38% of PBRM1‐BD4 variants bound the H3K14ac peptide with an EC_50_ similar to or better than that of WT PBRM1‐BD4 (Figure S3). Since most 2D tools are trained on similar datasets and statistical assumptions [37, 38, 71], these biases in data sources and data processing overestimate the number of PBRM1 ccRCC variants annotated as damaging to bromodomain stability and function [37, 38].

Based on computational and in vitro assessments of PBRM1 bromodomain stability and binding to H3K14ac peptides, the 18 “damaging” PBRM1‐BD2 and ‐BD4 missense variants likely contribute to ccRCC by disrupting PBRM1 bromodomain stability and/or ligand binding. However, “VUS” variants are still capable of contributing to ccRCC. For example, the ccRCC variant M523I was previously stably expressed in U2OS sarcoma cells but was incapable of performing native PBRM1 functions, including attenuating transcription and facilitating DSB repair [26]. Consistent with these results, we observe that M523I (a variant annotated as “VUS” relative to impacts on bromodomain stability and ligand binding) is still relatively thermostable (*T*_*m*_52.2^°^C ± 0.2^°^C, Table S3), yet binds H3K14ac peptides with an EC_50_ ~4‐fold weaker than WT PBRM1‐BD4 (Figure S3 and Table S6). Therefore, as we have observed previously [23], the impacts of PBRM1 missense variants on bromodomain structure and function can alter the function of the entire PBRM1 protein as an epigenetic Kac “reader” [17, 23, 24, 47] and tumor suppressor in ccRCC [20, 23–25]. Strikingly, molecular‐scale perturbations directly correlate with the outcomes of patients with cRCC; by mining publicly available data from The Cancer Genome Atlas ccRCC dataset [13, 98], we determined that IMFS for PBRM1‐BD2 and ‐BD4 missense variants (e.g., damaging protein impacts) exhibit an improved correlation with overall survival in patients with ccRCC (*r* ^2^ = 0.65) compared to 2D scores alone (*r* ^2^ = 0.51) (Figure 6E,F).

Although the correlation between 2D scores and biophysical metrics did not substantially improve when 3D and 4D scores were incorporated into IMFS, PCA shows that IMFS appropriately distinguishes population variants from ccRCC variants compared with 2D scores alone (Figures 5 and 6). Therefore, each level of the structural genomics analysis provides complementary data to improve the impact annotation of PBRM1 ccRCC variants. 2D impact scores provide quick estimates of missense variant molecular fitness, while 3D and 4D scores provide key structural and functional information to accelerate the mechanistic interpretation of missense variant molecular fitness impacts.

We acknowledge the limitations of the implicit distance‐dependent dielectric solvent model and the 10 ns timescale used in our MD simulations, as the short timeframe and the absence of explicit water molecules may affect the calculated parameters that contribute to 3D/4D scores [99]. Longer MD simulations with explicit solvent models enable a more detailed calculation of these parameters [99]. However, MD simulations using implicit solvent models and shorter time scales are faster and less computationally expensive than those using explicit solvent models [99]. Moreover, MD simulations lasting tens of nanoseconds are often used to sample protein conformations in equilibrium [100]. The overall goal of employing 10 ns MD simulations run under the implicit distance‐dependent dielectric solvent model in our multitiered structural genomics analysis is to accelerate clinical interpretation of missense variants and improve precision medicine initiatives in ccRCC and other diseases; longer, more computationally intensive simulations with explicit solvent models do not contribute towards this goal. Accordingly, the 3D and 4D levels of our integrated computational analysis serve as comparative screening tools to rank variant impacts rather than quantitatively precise absolute free energy predictions, which would require extensive conformational sampling and explicit solvent modeling beyond the scope of this study; nonetheless, these comparative metrics successfully distinguish population variants from ccRCC variants and correlate with experimental outcomes.

The majority of variants analyzed in this study are found in patients with confirmed ccRCC cases. However, the PBRM1 ccRCC variants that we annotated as biophysically “tolerated” vis‐à‐vis bromodomain stability and ligand binding do not indicate a lack of contribution to ccRCC pathobiology. Instead, biophysically “tolerated” missense variants may disrupt other PBRM1 functions that can be probed in future studies, such as interactions with nonhistone proteins (e.g., p53) [20] or DSB repair [26]. The interaction specificity of “reader” domains for epigenetic histone modifications can also differ in the setting of whole nucleosomes versus short histone peptides [101–103]. Accordingly, the impacts of PBRM1 ccRCC variants on H3K14ac peptide binding that we observed here (Figure 4, Figure S3, and Table S6) may be altered in the setting of nucleosomes containing H3K14ac. Therefore, additional investigations are required to assess how PBRM1 ccRCC variants may contribute to alternative mechanisms of disrupted epigenetic homeostasis in ccRCC.

## 5. Conclusions

The findings of the current study demonstrate that integrating 3D structural and 4D functional assessments with 2D sequence‐based variant impact tools refines the annotation of PBRM1 bromodomain missense variants in ccRCC. The additional information provided by 3D and 4D metrics in IMFSs generates hypotheses that reflect the mechanisms by which variant impacts affect bromodomain structural integrity and Kac binding. Notably, IMFSs allow annotation of PBRM1 missense variants found in the general population or in ccRCC patients in a manner more reflective of biophysical PBRM1 missense variant stability and ligand binding compared to 2D variant impact scores or even individual 2D algorithms (i.e., AlphaMissense and ESM1b). Taken together, we find that PBRM1‐BD2 and ‐BD4 missense variants exhibit a wide range of molecular fitness, with predicted and observed impacts on bromodomain stability, structural integrity, and ligand binding. As a result, patients with biophysically “tolerated” missense variants may have poorer therapeutic responses to antiangiogenics and ICB, similar to patients with WT PBRM1 protein expression [27–31]. In contrast, patients with biophysically “damaging” missense variants may have improved responses to ccRCC therapeutics, phenocopying patients with complete loss of PBRM1 protein expression [27–31]. Therefore, this study provides actionable assessments of the impacts of PBRM1 missense variants on protein function, supporting future studies that correlate specific ccRCC PBRM1 missense variants with altered PBRM1 cellular functions and therapeutic responses to inform personalized medicine approaches in ccRCC.

## Funding

The study was funded by the National Institute of General Medical Sciences (10.13039/100000057, R35GM128840, T32GM154638, and P41GM111135) and the National Cancer Institute (10.13039/100000054, F30CA278386).

## Disclosure

The results shown here are in part based upon data generated by the TCGA Research Network: https://cancergenome.nih.gov/ [13, 98].

## Conflicts of Interest

The authors declare no conflicts of interest.

## Supporting information


**Supporting Information** Additional supporting information can be found online in the Supporting Information section. These files contain Supporting Materials and Methods, Tables S1–S9, and Figures S1–S8.

## Data Availability

The data that support the findings of this study are available from the corresponding author upon reasonable request.
