# Supplementary material for: Structural Genomics Defines PBRM1 Bromodomain Variant Function in ccRCC
Source: Hum Mutat. 2026 Aug 5;2026:9214621. doi: 10.1155/humu/9214621 (PMC13439403; doi:10.1155/humu/9214621)
Supplement: Supplementary file 1 [file HUMU-2026-9214621-s001.zip › Supporting Information Human Mutation.pdf]

Structural genomics defines PBRM1 bromodomain variant function in ccRCC

Karina L. Bursch,[1,2] Salomão D. Jorge,[2] Audrey E. Catlin,[1] Raymundo Nuñez,[1,2] Michael D. LeClaire,[1,2] Jacob Licklider,[2] Raul A. Urrutia,[1,2,3] and Brian C. Smith[1,2,4]

[1]Department of Biochemistry, Medical College of Wisconsin, Milwaukee, WI 53226, USA; [2]Structural Genomics Unit, Linda T. and John A. Mellowes Center for Genomic Sciences and Precision Medicine, Medical College of Wisconsin, Milwaukee, WI 53226, USA; [3]Department of Surgery, Medical College of Wisconsin, Milwaukee, WI 53226, USA; [4]Program in Chemical Biology, Medical College of Wisconsin, Milwaukee, WI 53226, USA

For correspondence: Brian C. Smith, brismith@mcw.edu

Table of Contents

SUPPLEMENTAL MATERIALS AND METHODS 4

SUPPLEMENTAL TABLES 8

Table S1. Scores for the 48 sequence-based algorithms used at the 2D level of the integrated computational analysis. 8

Table S2. Raw values, Z scores, and scaled Z scores for the 7 structure-based metrics and the 7 molecular dynamics-based impact metrics used at the 3D and 4D levels of the integrated computational analysis. 8

Table S3. PBRM1-BD2 and -BD4 wild-type and missense variant T_m and ΔT_m values. 9

Table S4. PBRM1-BD2 and -BD4 wild-type and missense variant AlphaScreen counts. 10

Table S5. Percent contributions of integrated structural genomics analysis metrics to the first two components of PBRM1-BD2 and PBRM-BD4 missense variant PCA. 11

Table S6. AlphaScreen EC_{50} values and fold changes relative to wild-type PBRM1-BD2 and -BD4. 13

Table S7. Sensitivity and specificity from ROC with IMFS and H3K14ac peptide binding. 14

Table S8. Scores for the 44 2D metrics, five 3D metrics, and two 4D metrics from the final integrated computational analysis. 15

Table S9. Sensitivity and specificity from ROC with 3D/4D score absolute difference and H3K14ac peptide binding. 15

SUPPLEMENTAL FIGURES 16

Figure S1. Circular dichroism spectra of PBRM1-BD2 and -BD4 population and ccRCC-associated missense variants. 16

Figure S2. ^{1}H NMR spectra of PBRM1-BD2 and -BD4 population and ccRCC-associated missense variants. 17

Figure S4. Electrophoretic mobility shift assays of PBRM1-BD2 and PBRM1-BD4 wild-type constructs with and without a His_6 tag, and PBRM1-BD2 and PBRM1-BD4 population and ccRCC-associated missense variants (0-50 µM protein) binding to Widom 601 DNA (150 nM). 19

Figure S6. Correlation of biophysical data with individual metrics across the initial integrated computational analysis. 22

Figure S7. Soluble and insoluble PBRM1-BD2 and -BD4 ccRCC variants cluster separately from population variants. 23

Figure S8. ROC analyses for score threshold determination. 24

REFERENCES 25

Supplemental Materials and Methods

Site-directed mutagenesis. The His_6-tagged PBRM1-BD2 bromodomain construct (aa 175-291) in the pNIC28-Bsa4 vector and the His_6-tagged PBRM1-BD4 bromodomain construct (aa 496–637) in the pNIC-CTHF vector were gifts from Nicola Burgess-Brown (Addgene plasmids #39013 and #39103, respectively). Cancer-associated and population missense variants of PBRM1-BD2 and -BD4 were generated by site-directed mutagenesis (GenScript) and confirmed by Sanger sequencing.

Protein expression and purification. Recombinant PBRM1-BD2 WT, PBRM1-BD4 WT, and population and ccRCC-associated PBRM1-BD2/4 missense variants were purified from BL21(DE3) *E. coli* by nickel-affinity chromatography as previously described.[1] BL21(DE3) cells were transformed and grown at 37 °C in 2XYT or Terrific Broth medium with 50 mg/L kanamycin to an optical density of 0.4-0.6 at 600 nm. Bacterial cultures were then grown at 18 °C to an optical density of 0.6-0.8 at 600 nm. Protein expression was then induced overnight with 0.1 mM IPTG at 18 °C. Cells were harvested *via* centrifugation at 5,000 rpm, and cell pellets were frozen at -80 °C until lysis. Frozen cells were thawed on ice and resuspended in lysis buffer (50 mM HEPES, 500 mM NaCl, 5% v/v glycerol, 5 mM imidazole, pH 7.5). 0.3 nM aprotinin, 1 nM E-64, 1 nM leupeptin, 1 nM bestatin, 1 nM pepstatin A, and 100 nM PMSF protease inhibitors were added to the lysis buffer used to resuspend frozen cells, along with 5 µg/mL DNase I and 5 mM $MgCl_2$. Resuspended cells were immediately lysed via sonication for 10 minutes (pulsed, amplitude 3.5, 50% work cycle), and lysates were clarified by centrifugation for 30 minutes at 30,000 rpm. Clarified lysates were then applied to nickel (Ni)-charged MagBeads (GenScript, L00295) (~1 mL beads/L culture) at 4 °C overnight while the samples were rocked. The protein-bound Ni-charged MagBeads were washed with 3 × 10 mL lysis buffer containing 0.02% v/v Tween 20, followed by 3 × 10 mL lysis buffer alone. Protein was eluted off the beads with 5 mL elution buffer (50 mM HEPES, 500 mM NaCl, 5% v/v glycerol, 150 mM imidazole, pH 7.5) using the AmMag SA Plus semiautomated purification system (GenScript, L01013). For select missense variants, clarified lysates were applied to nickel-nitrilotriacetic acid (Ni-NTA) resin (~1 mL resin/L culture) at 4 °C overnight while rocking. The protein-bound Ni-NTA resin was applied to a column and washed with 15 mL fractions of lysis buffer until nonspecific protein elution had ceased, as determined by monitoring the absorbance of the collected wash fractions at 280 nm. Protein was then eluted from the Ni-NTA resin using increasing concentrations of imidazole in lysis buffer (5 mL of 50, 100, 150, 200, and 250 mM imidazole). Fractions were resolved by SDS-PAGE, and those containing the PBRM1 bromodomain of interest were pooled. Protein

samples were further purified by gel filtration using an Enrich SEC 70 10 × 300 mm column (Bio-Rad), a Superdex 75 Increase 10/300 GL column (Cytiva, 29148721), a Superdex 200 Increase 10/300 GL column (Cytiva, 28990944) an XK 16/40 column (Cytiva, 28988938) packed with Sephacryl S-200 resin (Cytiva, 17-0584-01), or a HiLoad 16/600 Superdex 75 column (Cytiva, 28989333) into storage buffer (50 mM HEPES, 500 mM NaCl, 5% v/v glycerol, pH 7.5) or EMSA buffer (50 mM K_3PO_4, 50 mM KCl, 5% v/v glycerol, 1 mM DTT, pH 7). Monomeric protein was collected based on the chromatographs resulting from gel-filtration size-exclusion chromatography. Concentrations of purified proteins were determined by the method of Bradford using bovine serum albumin as a standard,[2] aliquoted, flash-frozen, and stored at -80 °C.

Nano-differential scanning fluorimetry (nanoDSF). nanoDSF was performed as previously described.[1] PBRM1-BD2 or -BD4 proteins were prepared at a concentration of 30 µM in EMSA buffer (50 mM K_3PO_4, 50 mM KCl, 5% v/v glycerol, 1 mM DTT, pH 7). Samples were drawn up in triplicate in Prometheus NT.48 high-sensitivity capillaries (NanoTemper Technologies, PR-C006) and run at 100% excitation power with a temperature ramp from 20 °C to 95 °C, increasing by 1 °C/min, using a Prometheus NT.48 instrument (PR001). The intrinsic fluorescence ratio (350 nm/330 nm) was plotted versus temperature, and PBRM1-BD2 and -BD4 protein T_m was determined by identifying the maximum or minimum of the first derivative of the fluorescence signal with ThermControl software version 2.3.1 (NanoTemper Technologies, https://nanotempertech.com/prometheus/nt48-software), where the associated error is standard deviation.

Circular dichroism. Circular dichroism was performed as previously described.[1] PBRM1-BD2 or -BD4 wild-type and missense variants were dialyzed into CD/NMR buffer (50 mM K_3PO_4, 50 mM NaF, 1 mM DTT, pH 6.8), gently stirring overnight at 4 °C using 3.5 kDa molecular weight cutoff Slide-a-lyzer mini dialysis units (Thermo Fisher Scientific, 69550). PBRM1-BD2 and -BD4 secondary structure was assessed using a J-1500 CD spectrophotometer (JASCO). The samples were prepared at a concentration of 0.01 – 0.02 mg/mL in CD buffer without DTT and placed in 10 mm quartz cuvettes (Thermo Fisher Scientific). CD spectra were recorded at 25 °C from 280 to 190 nm, with a data pitch of 0.1 nm. A bandwidth of 1 nm was used with a digital integration time of 1 s and a scanning speed of 50 nm/min. Each spectrum was accumulated from five scans and corrected by subtracting the buffer spectrum from the sample spectrum. Missense variant spectra were corrected by multiplying the CD signal by the ratio of the wild-

type bromodomain absorbance to the missense variant absorbance. The data were processed using Spectra Analysis software supplied by the manufacturer (JASCO, https://jascoinc.com/products/spectroscopy/molecular-spectroscopy-software/) and transferred to GraphPad Prism for presentation.

Nuclear magnetic resonance spectroscopy. NMR spectroscopy was performed as previously described.[1] PBRM1-BD2 and -BD4 wild-type and missense variants were dialyzed into CD/NMR buffer (50 mM K_3PO_4, 50 mM NaF, 1 mM DTT, pH 6.8), gently stirring overnight at 4 °C using 3.5 kDa molecular weight cutoff Slide-a-lyzer mini dialysis units (Thermo Fisher Scientific, 69550). PBRM1-BD2 and -BD4 proteins were prepared at a concentration of 100 μM in CD/NMR buffer supplemented with 0.2% w/v sodium azide and 5% v/v D_2O. ^1H NMR data were collected at 25 °C on a Bruker Avance II 600 MHz spectrometer equipped with a triple resonance *z*-axis gradient cryoprobe and SampleJet autosampler, which allowed for automatic tuning, matching, and shimming for each sample. ^1H-NMR experiments consisted of 128 scans for each protein construct. Spectra were processed with MNova from NMRBox (https://mestrelab.com/software/mnova-software/).[3]

AlphaScreen assays. Histone peptide AlphaScreen assays were performed as previously described.[1] All binding assays were conducted in light gray, half-area 96-well plates (PerkinElmer, 6002350) in a total volume of 40 μL. All stock solutions were prepared in the assay buffer, which comprised 1× AlphaLISA Epigenetics buffer (Revvity, 5× AL008C/F), supplemented with 0.05% v/v Tween 20 and 1 mM tris(2-carboxyethyl)phosphine. For initial screens, each recombinant His_6-tagged PBRM1-BD2 or -BD4 WT or missense variant was incubated at three concentrations (0.1, 1, and 10 μM) with 100 nM biotinylated histone H3K14ac peptide (amino acid residues 1–20; H_2N-RTKQTARKSTGGK(acetyl)APRKQLK(biotin)-$CONH_2$; Peptide 2.0) in technical triplicates. For further testing of missense variants exhibiting peptide binding in the initial screens, each recombinant His_6-tagged PBRM1-BD2 or PBRM1-BD4 missense variant was incubated at a concentration range of 0.1-20 μM or 0.001-2 μM with 100 nM biotinylated histone H3K14ac peptide. A 4× (400 nM) biotinylated histone H3K14ac peptide stock solution (10 μL) and 4× protein stock solutions (10 μL) were added to each well. The plate was then incubated at room temperature for 30 minutes. A bead solution comprising 8 μg/mL streptavidin donor beads and 8 μg/mL nickel-chelate acceptor beads (Revvity, AlphaScreen Histidine (Nickel Chelate) Detection Kit, 500 assay points, 6760619C) was prepared in assay buffer. 20 μL of bead solution was added to each well under reduced light, and the plate was

covered and incubated for an additional hour in the dark. Luminescence was subsequently read on a BioTek Cytation 5 imaging reader (Agilent, 16277) using the Alpha filter cube (Agilent, 1325000). The resulting Alpha counts were analyzed with GraphPad Prism. Alpha counts for initial screens were corrected by subtracting the Alpha counts from the average of the two negative control samples (100 nM peptide alone and 10 µM protein alone). Corrected Alpha counts of PBRM1-BD2/4 missense variants were then normalized to those of the wild-type bromodomains. Raw Alpha counts for titrations were fit to a sigmoidal non-linear regression, then normalized based on the bottom and top values of the fit.

Electrophoretic mobility shift assays. EMSAs were performed as previously described.[1] 5% 75:1 acrylamide:bisacrylamide native gels were cast and pre-run in chilled 0.2× Tris-borate-EDTA (TBE) buffer (diluted from 10× TBE buffer (BioRad, 1610770) with MilliQ H_2O), on ice for 60 min at 125 V. Samples were prepared by mixing 150 nM Widom 601 DNA (a gift from Dr. Emma Morrison, Department of Biochemistry, Medical College of Wisconsin[4]) with 0 to 50 µM PBRM1-BD2/4 WT or missense variants purified in EMSA buffer (final assay concentration 10 mM K_3PO_4, 10 mM KCl, 1% v/v glycerol, 0.2 mM DTT, pH 7). The samples were then mixed with an equivalent volume of 2× loading dye (10% w/v sucrose and 0.02% w/v bromophenol blue in 0.5× TBE buffer (diluted from 10× TBE buffer (BioRad, 1610770) with MilliQ H_2O) and equilibrated on ice for 1 hour. Samples were loaded onto the pre-run native gels in chilled 0.2× TBE buffer on ice. Gels were run for 45 to 60 minutes at 125 V. Gels were stained with 5 mL of 0.5 µg/mL ethidium bromide for 5 minutes, and bands were visualized using a ChemiDoc MP imaging system (Bio-Rad).

Supplemental Tables

Table S1. Scores for the 48 sequence-based algorithms used at the 2D level of the integrated computational analysis.

Table S2. Raw values, Z scores, and scaled Z scores for the 7 structure-based metrics and the 7 molecular dynamics-based impact metrics used at the 3D and 4D levels of the integrated computational analysis.

Table S3. PBRM1-BD2 and -BD4 wild-type and missense variant T_m and ΔT_m values.

**Protein**	**T_m (°C)**	**ΔT_m (°C)**
WT BD2	45.8 ± 0.4	NA
N263A	46.6 ± 0.1	0.7 ± 0.3
I193V	45.7 ± 0.9	-0.2 ± 1.3
N198S	47.1 ± 1.5	1.2 ± 1.9
H246R	43.1 ± 2.3	-2.7 ± 2.7
I204L	43.2 ± 0.01	-2.7 ± 0.4
S205C	45.9 ± 0.1	0.04 ± 0.3
Q238H	45.1 ± 0.2	-0.7 ± 0.2
N258I	32.1 ± 0.4	-13.7 ± 0.6
Y281F	39.7 ± 1.0	-6.2 ± 1.0
**Protein**	**T_m (°C)**	**ΔT_m (°C)**
WT BD4	55.5 ± 0.1	NA
N577S	53.4 ± 0.3	-2.1 ± 0.2
K591T	54.8 ± 0.9	-0.7 ± 0.9
R622K	54.9 ± 0.2	-0.6 ± 0.2
M523I	52.2 ± 0.2	-3.3 ± 0.1
L526S	31.5 ± 0.03	-24.0 ± 0.1
S538L	41.9 ± 0.1	-13.6 ± 0.1
S538P	43.3 ± 0.1	-12.1 ± 0.2
C543W	37.7 ± 0.2	-17.8 ± 0.2
P550L	≤20	≤-35.5
N574Y	46.3 ± 2.2	-9.1 ± 2.1
E583D	46.5 ± 0.1	-8.9 ± 0.2
I587T	43.4 ± 0.9	-12.0 ± 0.8
D610E	45.7 ± 0.2	-9.8 ± 0.2
L614V	44.3 ± 1.2	-11.2 ± 1.2
L618H	32.0 ± 0.1	-23.4 ± 0.2
G626V	48.4 ± 0.1	-7.0 ± 0.1

Values were determined by nanoDSF, where error represents standard deviation from samples run in technical triplicate. NA = value not applicable.

Table S4. PBRM1-BD2 and -BD4 wild-type and missense variant AlphaScreen counts.

[Protein], μM	Normalized AlphaCounts (10 μM protein)	Normalized AlphaCounts (1 μM protein)	Normalized AlphaCounts (0.1 μM protein)
N263A	ND	ND	ND
I193V	1.0 ± 0.05	1.3 ± 1.3	0.5 ± 0.4
N198S	1.0 ± 0.1	1.5 ± 0.5	55 ± 29
H246R	1.1 ± 0.04	1.7 ± 0.3	0.3 ± 0.1
I204L	1.1 ± 0.1	0.2 ± 0.1	ND
S205C	1.0 ± 0.1	1.5 ± 0.1	0.1 ± 1.1
Q238H	0.9 ± 0.1	1.1 ± 0.2	1.3 ± 0.3
N258I	ND	ND	0.1 ± 0.2
Y281F	1.2 ± 0.03	0.6 ± 0.1	ND
**[Protein], μM**	**Normalized AlphaCounts (10 μM protein)**	**Normalized AlphaCounts (1 μM protein)**	**Normalized AlphaCounts (0.1 μM protein)**
N577S	1.2 ± 0.1	1.1 ± 0.1	1.0 ± 0.7
K591T	1.1 ± 0.1	1.2 ± 0.1	1.8 ± 0.2
R622K	1.2 ± 0.1	1.2 ± 0.1	1.6 ± 0.3
M523I	0.6 ± 0.1	1.0 ± 0.02	2.1 ± 0.2
L526S	0.9 ± 0.04	0.4 ± 0.04	ND
S538L	ND	0.6 ± 0.3	1.2 ± 0.3
S538P	1.3 ± 0.1	1.3 ± 0.1	0.9 ± 0.2
C543W	ND	0.7 ± 0.2	0.1 ± 0.1
P550L	ND	ND	ND
N574Y	1.1 ± 0.1	1.4 ± 0.3	1.8 ± 1.1
E583D	1.1 ± 0.1	1.1 ± 0.1	1.3 ± 0.6
I587T	1.1 ± 0.1	1.1 ± 0.1	1.4 ± 0.5
D610E	1.1 ± 0.1	1.4 ± 0.6	1.1 ± 1.8
L614V	1.2 ± 0.1	1.4 ± 0.5	ND
L618H	1.1 ± 0.1	1.4 ± 0.6	3.4 ± 5.0
G626V	1.0 ± 0.1	1.4 ± 0.7	5.7 ± 7.7

Error represents the standard deviation from n=3 experimental replicates. ND = normalized AlphaCounts value was not detectable (< 0.05).

Table S5. Percent contributions of integrated structural genomics analysis metrics to the first two components of PBRM1-BD2 and PBRM-BD4 missense variant PCA.

Analysis Metric	PC1 (%)	PC2 (%)
2D SIFT	0.12	0.04
2D SIFT4G	0.16	0.06
2D Polyphen2 HDIV	0.17	0.06
2D Polyphen2 HVAR	0.18	0.06
2D LRT	0.07	0.01
2D MutationTaster	0.14	0.08
2D MutationAssessor	0.16	0.00
2D FATHMM	0.05	0.00
2D PROVEAN	0.17	0.05
2D VEST4	0.20	0.02
2D MetaSVM	0.16	0.00
2D MetaLR	0.21	0.02
2D MetaRNN	0.23	0.06
2D MCAP	0.21	0.02
2D REVEL	0.22	0.05
2D MVP	0.12	0.00
2D MPC	0.15	0.02
2D PrimateAI	0.16	0.03
2D DEOGEN2	0.15	0.03
2D BayesDel_addAF	0.19	0.16
2D BayesDel_noAF	0.18	0.15
2D ClinPred	0.21	0.08
2D LIST_S2	0.03	0.04
2D CADD	0.06	0.30
2D CADD_hg19	0.12	0.22
2D fathmm_MKL_coding	0.03	0.21
2D fathmm_XF_coding	0.08	0.15
2D Eigen_raw_coding	0.13	0.21
2D Eigen_PC_raw_coding	0.10	0.25
2D GenoCanyon	0.04	0.08
2D HUVEC fitCons	0.01	0.02
2D GERP_RS	0.00	0.11
2D phyloP100way_vertebrate	0.03	0.18
2D phyloP470way_mammalian	0.02	0.17
2D phyloP17way_primate	0.00	0.20

2D phastCons17way_primate	0.00	0.09
2D SiPhy_29way_logOdds	0.05	0.01
2D gMVP	0.21	0.04
2D VARITY_R	0.19	0.05
2D VARITY_ER	0.20	0.06
2D VARITY_R_LOO	0.19	0.05
2D VARITY_ER_LOO	0.20	0.06
2D ESM1b	0.17	0.04
2D AlphaMissense	0.20	0.05
2D scores	9.00	4.92
3D $\Delta\Delta G_{folding\text{-}Ala}$	0.07	0.06
3D $\Delta\Delta G_{folding\text{-}FoldX}$	0.55	2.25
3D $\Delta\Delta G^{folding\text{-}CHARMM}$	0.35	0.26
3D ΔLocal 10Å RMSD	0.93	0.04
3D ΔGlobal RMSD	0.46	0.39
3D scores	5.33	6.52
4D $\Delta\Delta G_{MM\text{-}PBSA}$	2.79	0.56
4D $\Delta\Delta G_{CIE}$	3.97	0.05
4D scores	4.68	0.66
3D/4D scores	8.52	0.23
IMFS	12.05	4.77
ΔT_m	6.05	8.02
$CD_{222\ nm}$	4.10	22.84
$CD_{208\ nm}$	4.88	20.50
$NMR_{(0\text{-}1.25\ ppm)}$	3.84	2.97
$NMR_{(6.5\text{-}9.5\ ppm)}$	3.29	18.02
$NAC_{10\ \mu M}$	9.98	0.28
$NAC_{1\ \mu M}$	7.88	0.47
$NAC_{0.1\ \mu M}$	2.33	1.42
$NAC_{0.1\text{-}10\ \mu M}$	3.30	1.18

NAC= normalized AlphaCounts

Table S6. AlphaScreen EC_{50} values and fold changes relative to wild-type PBRM1-BD2 and -BD4.

WT EC_{50} (μM)	Missense variant	EC_{50} (μM)	FC relative to WT
0.4 ± 0.3	I193V	0.5 ± 0.3	0.8
0.4 ± 0.1	N198S	0.1 ± 0.02	4
0.4 ± 0.3	H246R	0.4 ± 0.2	1
1.1 ± 0.2	I204L	11.1 ± 1.0	0.10
1.7 ± 0.4	S205C	1.8 ± 0.5	0.94
1.8 ± 1.1	Q238H	2.1 ± 1.2	0.86
0.4 ± 0.1	Y281F	0.7 ± 0.1	0.57
**WT EC_{50} (μM)**	**Missense variant**	**EC_{50} (μM)**	**FC relative to WT**
0.2 ± 0.02	N577S	0.1 ± 0.02	2
0.2 ± 0.03	K591T	0.1 ± 0.02	2
0.3 ± 0.1	R622K	0.1 ± 0.01	3
0.4 ± 0.02	M523I	0.1 ± 0.01	4
0.1 ± 0.01	L526S	0.7 ± 0.1	0.14
0.4 ± 0.1	S538L	0.1 ± 0.02	4
0.9 ± 0.3	S538P	0.8 ± 0.2	1.13
0.1 ± 0.03	C543W	0.4 ± 0.1	0.25
0.4 ± 0.2	N574Y	0.1 ± 0.03	4
0.1 ± 0.02	E583D	0.1 ± 0.01	1
0.1 ± 0.02	I587T	0.1 ± 0.01	1
0.1 ± 0.01	D610E	0.3 ± 0.01	0.33
0.2 ± 0.2	L614V	0.5 ± 0.2	0.4
0.1 ± 0.02	L618H	0.2 ± 0.01	0.5
0.3 ± 0.2	G626V	0.1 ± 0.03	3

FC = fold change

Table S7. Sensitivity and specificity from ROC with IMFS and H3K14ac peptide binding.

Threshold	Sensitivity	Sensitivity 95% CI	Specificity	Specificity 95% CI	Youden Index (J)
0.21	1	0.78-1	0.06	0-0.27	0.05882
0.27	1	0.78-1	0.12	0.020-0.34	0.12
0.32	1	0.78-1	0.18	0.06-0.41	0.18
0.33	1	0.78-1	0.24	0.10-0.47	0.24
0.34	1	0.78-1	0.29	0.13-0.53	0.29
0.35	1	0.78-1	0.35	0.17-0.59	0.35
0.37	1	0.78-1	0.41	0.22-0.64	0.41
0.38	1	0.78-1	0.47	0.26-0.69	0.47
0.38	0.93	0.69-1	0.47	0.26-0.69	0.40
0.40	0.93	0.69-1	0.53	0.31-0.74	0.46
0.41	0.93	0.69-1	0.59	0.36-0.78	0.52
0.43	0.86	0.60-0.97	0.59	0.36-0.78	0.45
0.46	0.86	0.60-0.97	0.65	0.41-0.83	0.50
0.48	0.86	0.60-0.97	0.71	0.47-0.87	0.56
**0.48**	0.86	0.60-0.97	0.76	0.53-0.90	**0.62**
0.49	0.79	0.52-0.92	0.76	0.53-0.90	0.55
0.49	0.79	0.52-0.92	0.82	0.59-0.94	0.61
0.49	0.71	0.45-0.88	0.82	0.59-0.94	0.54
0.50	0.64	0.39-0.84	0.82	0.59-0.94	0.47
0.52	0.64	0.39-0.84	0.88	0.66-0.98	0.53
0.55	0.57	0.33-0.77	0.88	0.66-0.98	0.45
0.55	0.57	0.33-0.77	0.94	0.73-1	0.51
0.55	0.50	0.27-0.73	0.94	0.73-1	0.44
0.57	0.43	0.21-0.67	0.94	0.73-1	0.37
0.58	0.36	0.16-0.61	0.94	0.73-1	0.30
0.59	0.36	0.16-0.61	1	0.82-1	0.36
0.62	0.29	0.12-0.55	1	0.82-1	0.29
0.65	0.21	0.08-0.48	1	0.82-1	0.21
0.66	0.14	0.03-0.40	1	0.82-1	0.14
0.68	0.07	0-0.31	1	0.82-1	0.07

The optimal threshold and the Youden index are bolded.

Table S8. Scores for the 44 2D metrics, five 3D metrics, and two 4D metrics from the final integrated computational analysis.

Table S9. Sensitivity and specificity from ROC with 3D/4D score absolute difference and H3K14ac peptide binding.

Threshold	Sensitivity	Sensitivity 95% CI	Specificity	Specificity 95% CI	Youden Index (J)
0.02	0.86	0.49-0.99	0	0-0.49	-0.14
0.04	0.86	0.49-0.99	0.25	0.01-0.70	0.11
0.09	0.86	0.49-0.99	0.5	0.09-0.91	0.36
0.15	0.86	0.49-0.99	0.75	0.30-0.99	0.61
**0.17**	0.86	0.49-0.99	1	0.51-1	**0.86**
0.19	0.71	0.36-0.95	1	0.51-1	0.71
0.22	0.57	0.25-0.84	1	0.51-1	0.57
0.30	0.43	0.16-0.75	1	0.51-1	0.43
0.44	0.29	0.05-0.64	1	0.51-1	0.29
0.61	0.14	0.01-0.51	1	0.51-1	0.14

The optimal threshold and the Youden index are bolded.

Supplemental Figures

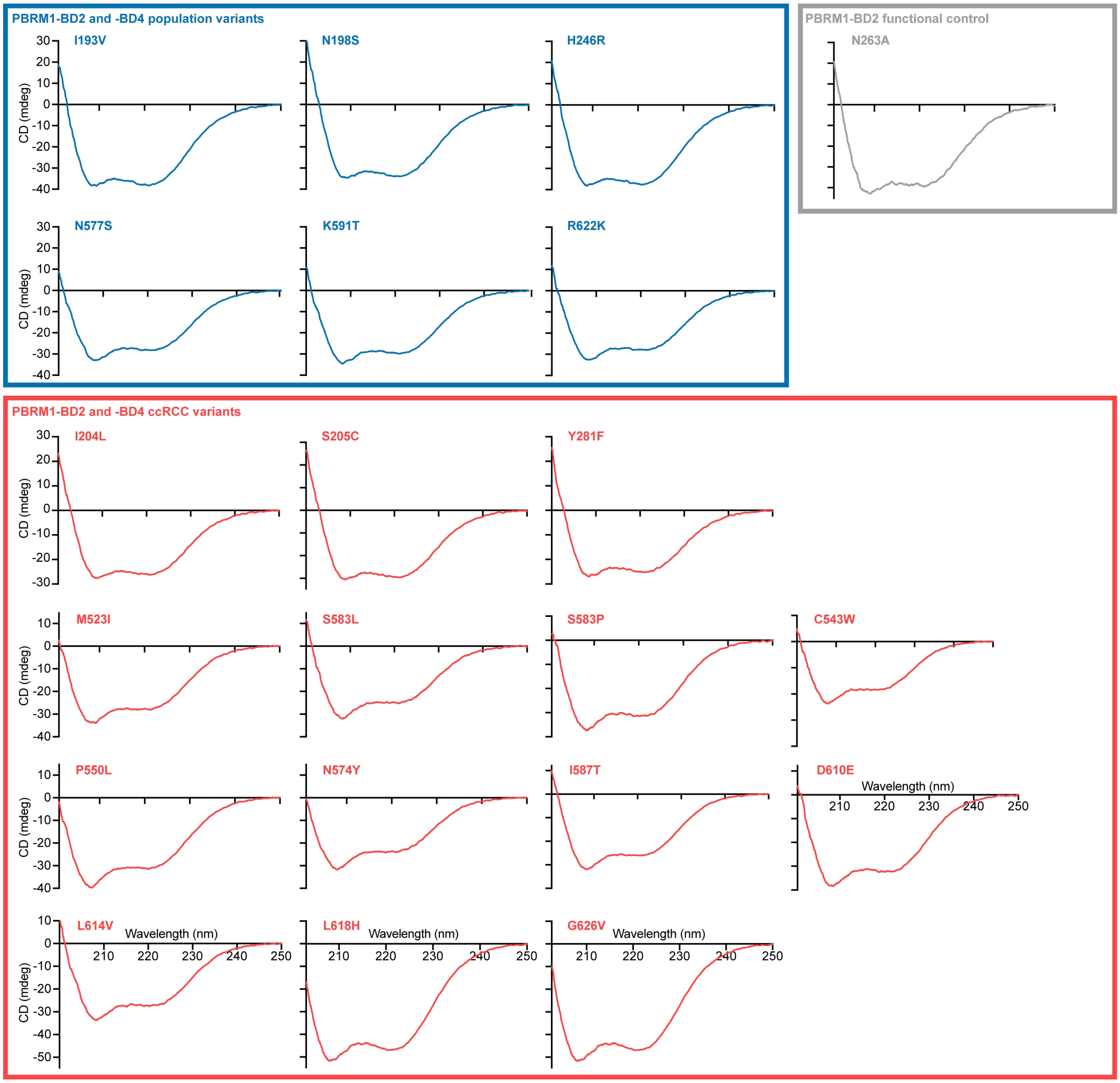

Figure S1. Circular dichroism spectra of PBRM1-BD2 and -BD4 population and ccRCC-associated missense variants.

Functional controls shown in gray, population variants in blue, ccRCC missense variants in red.

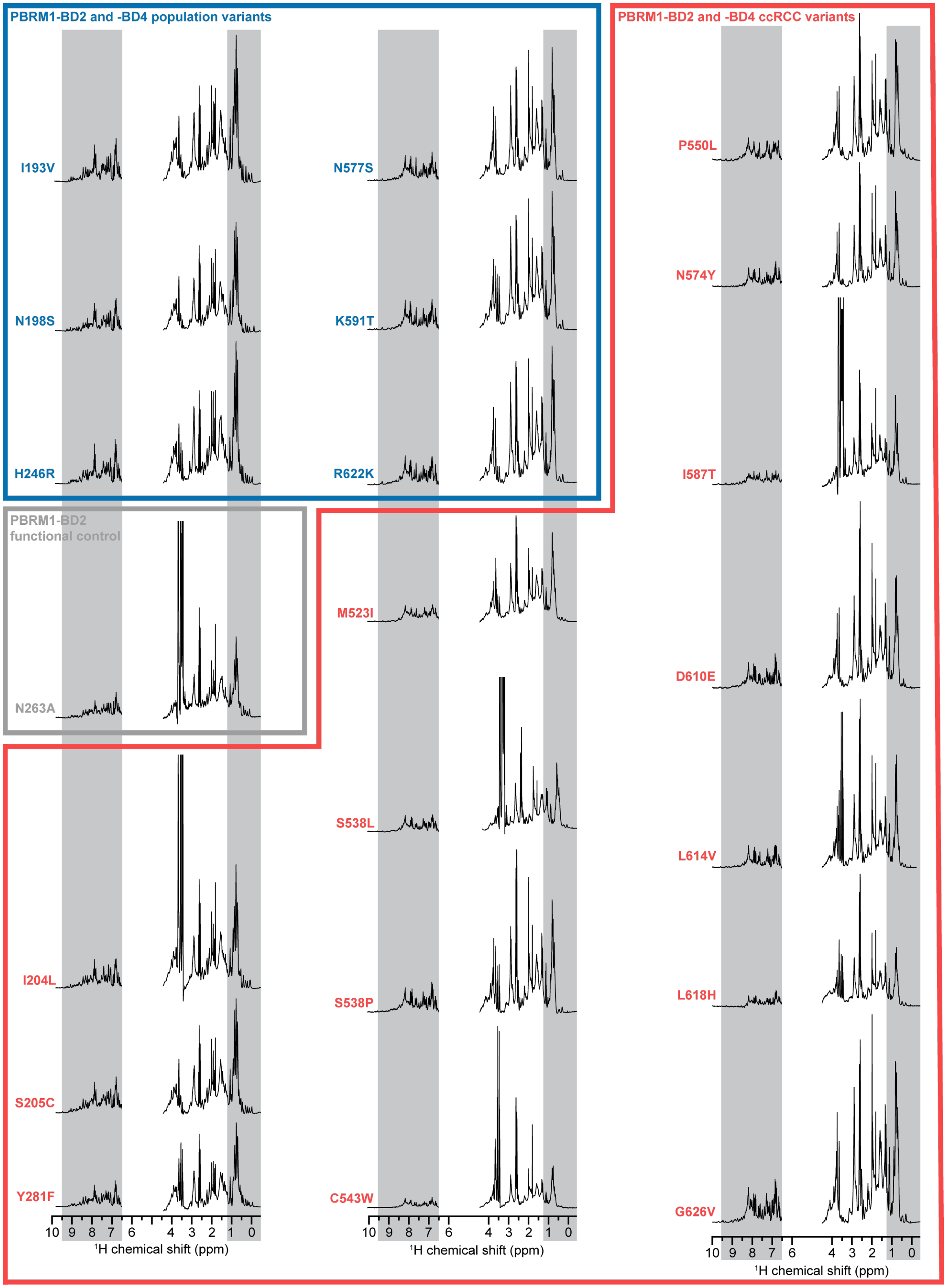

Figure S2. ^{1}H NMR spectra of PBRM1-BD2 and -BD4 population and ccRCC-associated missense variants.

Functional controls shown in gray, population variants in blue, ccRCC missense variants in red.

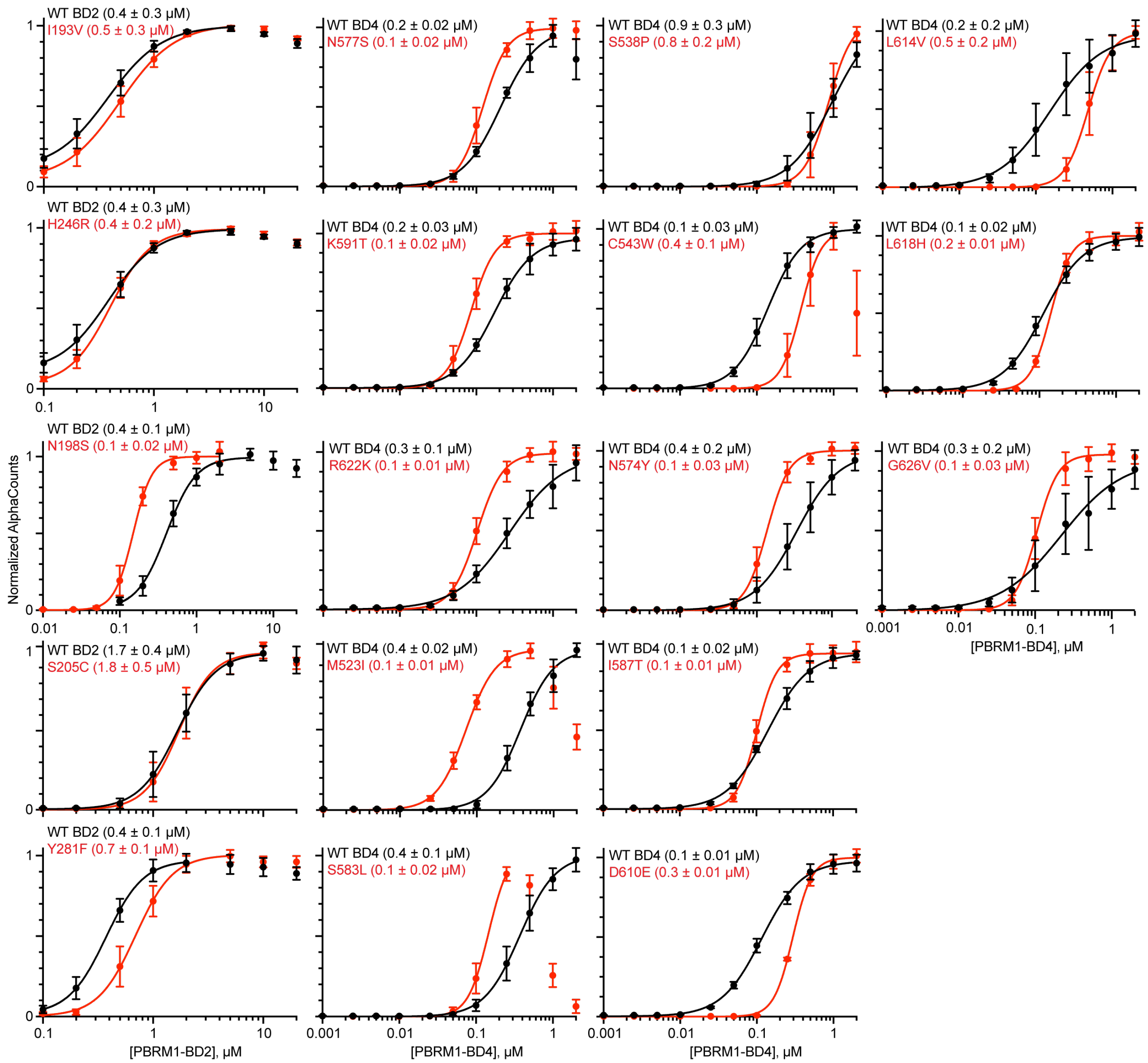

Figure S3. Half-maximal effective concentration (EC_{50}) of PBRM1-BD2 (0.1-20 μM) and PBRM1-BD4 (0.001-2 μM) missense variants binding to biotinylated H3K14ac peptide (100 nM).

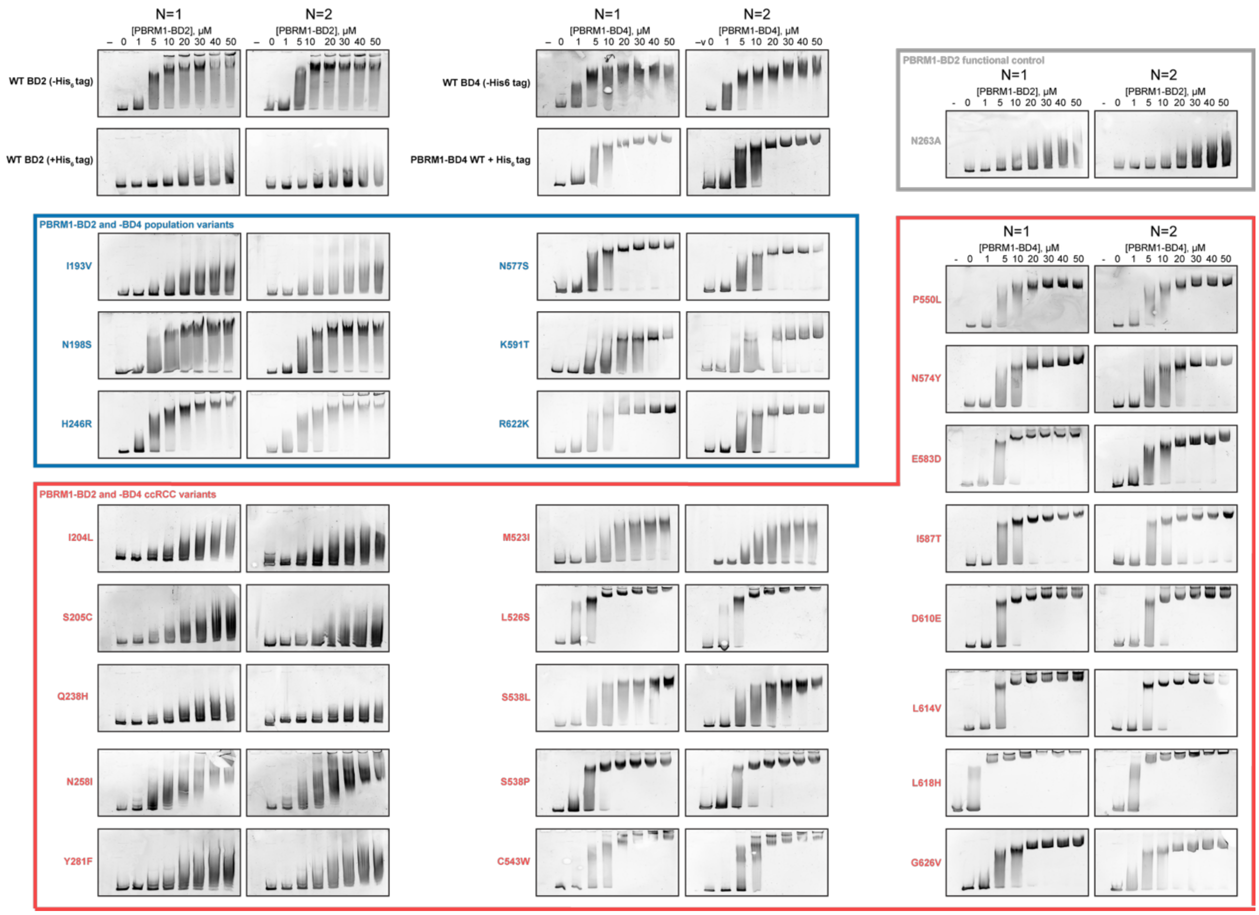

Figure S4. Electrophoretic mobility shift assays of PBRM1-BD2 and PBRM1-BD4 wild-type constructs with and without a His_6 tag, and PBRM1-BD2 and PBRM1-BD4 population and ccRCC-associated missense variants (0-50 μM protein) binding to Widom 601 DNA (150 nM). Functional controls shown in gray, population variants in blue, ccRCC missense variants in red.

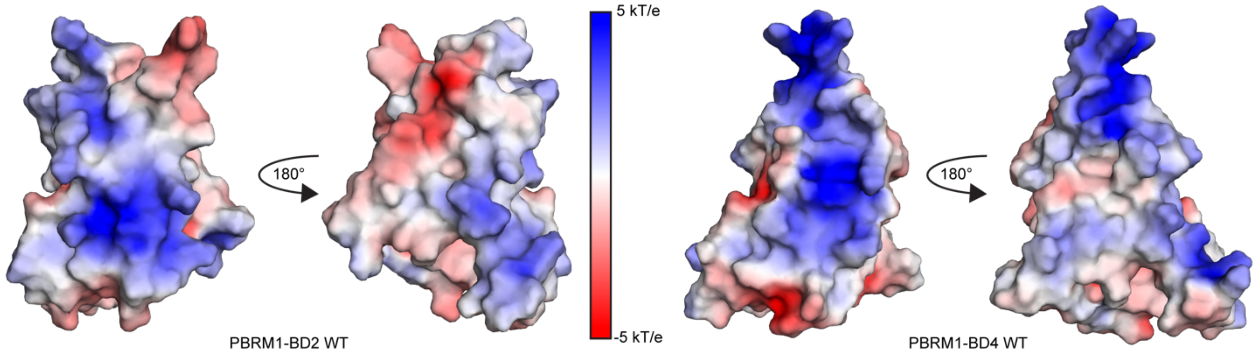

Figure S5. Electrostatic potential surface maps of wild-type PBRM1-BD2 and -BD4. Positive electrostatic potentials are colored blue, negative electrostatic potentials are colored red.

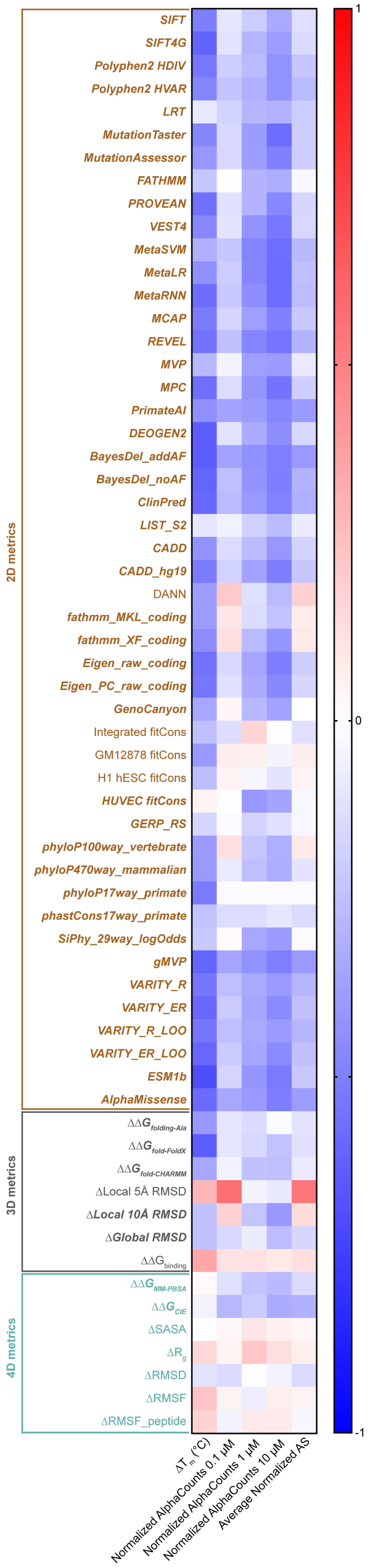

2D metrics
SIFT
SIFT4G
Polyphen2 HDIV
Polyphen2 HVAR
LRT
MutationTaster
MutationAssessor
FATHMM
PROVEAN
VEST4
MetaSVM
MetaLR
MetaRNN
MCAP
REVEL
MVP
MPC
PrimateAI
DEOGEN2
BayesDel_addAF
BayesDel_noAF
ClinPred
LIST_S2
CADD
CADD_hg19
DANN
fathmm_MKL_coding
fathmm_XF_coding
Eigen_raw_coding
Eigen_PC_raw_coding
GenoCanyon
Integrated fitCons
GM12878 fitCons
H1 hESC fitCons
HUVEC fitCons
GERP_RS
phyloP100way_vertebrate
phyloP470way_mammalian
phyloP17way_primate
phastCons17way_primate
SiPhy_29way_logOdds
gMVP
VARITY_R
VARITY_ER
VARITY_R_LOO
VARITY_ER_LOO
ESM1b
AlphaMissense
3D metrics
$\Delta\Delta G_{folding\text{-}Ala}$
$\Delta\Delta G_{fold\text{-}FoldX}$
$\Delta\Delta G_{fold\text{-}CHARMM}$
ΔLocal 5Å RMSD
ΔLocal 10Å RMSD
ΔGlobal RMSD
$\Delta\Delta G_{binding}$
4D metrics
$\Delta\Delta G_{MM\text{-}PBSA}$
$\Delta\Delta G_{CIE}$
ΔSASA
ΔR_g
ΔRMSD
ΔRMSF
ΔRMSF_peptide
ΔT_m (°C)
Normalized AlphaCounts 0.1 µM
Normalized AlphaCounts 1 µM
Normalized AlphaCounts 10 µM
Average Normalized AS
1
0
-1

Figure S6. Correlation of biophysical data with individual metrics across the initial integrated computational analysis.

2D metrics are colored brown, 3D metrics are colored gray, and 4D metrics are colored green, and each analysis level is separated by brackets. Metrics included in the final integrated structural genomics analysis are in **bold** and *italicized*. Average normalized AS compares the scoring metrics to the average normalized AlphaScreen counts across all protein concentrations tested in the initial AlphaScreens.

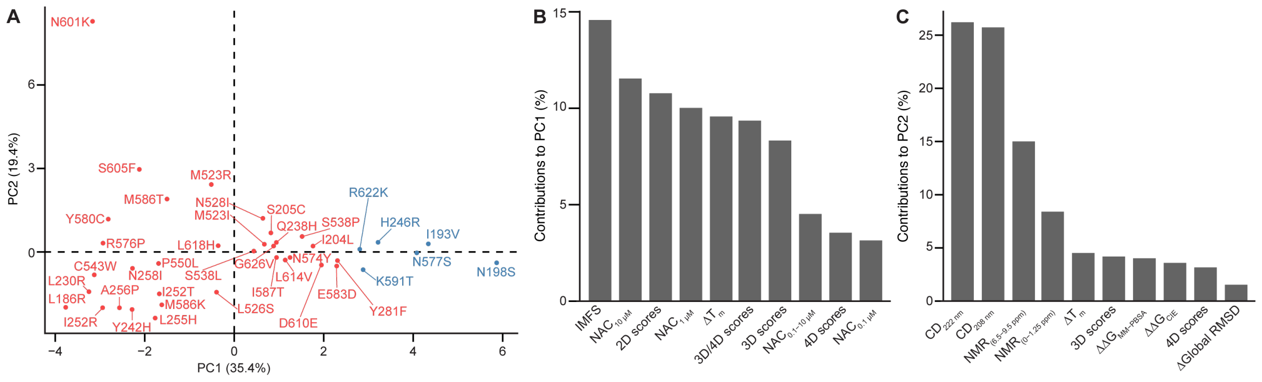

Figure S7. Soluble and insoluble PBRM1-BD2 and -BD4 ccRCC variants cluster separately from population variants.
(A) PCA of all PBRM1-BD2 and -BD4 variants with computational and biophysical data from the integrated structural genomics analysis as inputs for variant clustering, where missing biophysical data from insoluble variants is imputed as “worst case”. **(B)** Contribution of the top 10 metrics to the first principal component of the PCA plot. **(C)** Contribution of the top 10 metrics to the second principal component of the PCA plot.

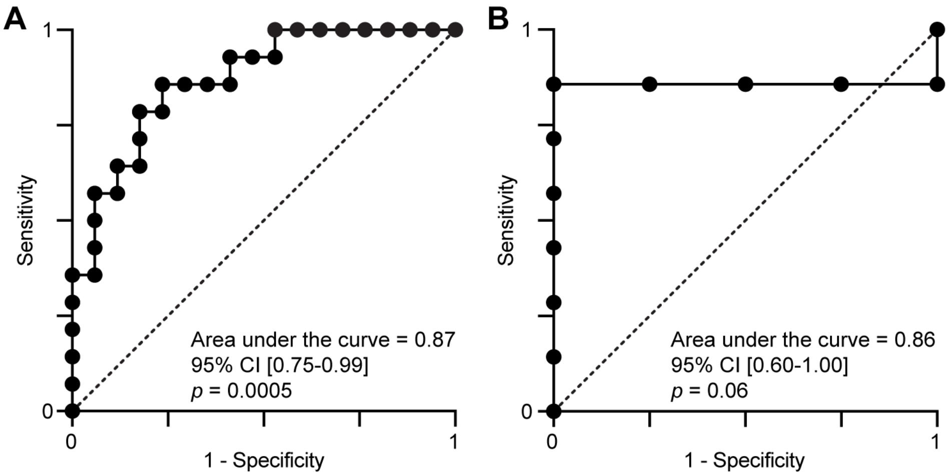

Figure S8. ROC analyses for score threshold determination.

(A) ROC analysis of all soluble PBRM1-BD2 and -BD4 variants based on IMFS and H3K14ac peptide binding (variant binding with normalized AlphaCounts ≥ 0.95) to determine the threshold for tolerated vs. VUS vs. damaging variant annotation. **(B)** ROC analysis of all soluble damaging PBRM1-BD2 and -BD4 variants based on absolute difference between 3D and 4D and H3K14ac peptide binding (variant binding with normalized AlphaCounts ≥ 0.01) to determine the threshold for SV vs. DV vs. SDV damaging variant sub-classification.

References

1. Bursch KL, Goetz CJ, Jiao G, Nuñez R, Olp MD, Dhiman A, Khurana M, Zimmermann MT, Urrutia RA, Dykhuizen EC, Smith BC. Cancer-associated polybromo-1 bromodomain 4 missense variants variably impact bromodomain ligand binding and cell growth suppression. J Biol Chem. 2024;300(4):107146. doi: 10.1016/j.jbc.2024.107146. PubMed PMID: 38460939; PMCID: PMC11002309.
2. Bradford MM. A rapid and sensitive method for the quantitation of microgram quantities of protein utilizing the principle of protein-dye binding. Analytical Biochemistry. 1976;72(1):248-54. doi: 10.1016/0003-2697(76)90527-3. PubMed PMID: 942051.
3. Maciejewski MW, Schuyler AD, Gryk MR, Moraru II, Romero PR, Ulrich EL, Eghbalnia HR, Livny M, Delaglio F, Hoch JC. NMRbox: A Resource for Biomolecular NMR Computation. Biophys J. 2017;112(8):1529-34. doi: doi.org/10.1016/j.bpj.2017.03.011; PMCID: PMC5406371.
4. Paintsil EA, Morrison EA. Preparation of Recombinant Histones and Widom 601 DNA for Reconstitution of Nucleosome Core Particles. Methods Mol Biol. 2023;2599:163-75. doi: 10.1007/978-1-0716-2847-8_12. PubMed PMID: 36427149.
